# Supplementary material for: Factors associated with severe COVID-19 outcomes among adults with at least a primary vaccination schedule: A retrospective cohort study from Alberta, Canada
Source: PLoS One. 2026 Aug 17;21(8):e0354822. doi: 10.1371/journal.pone.0354822 (PMC13480603; doi:10.1371/journal.pone.0354822)
Supplement: Supplementary file 1 [file pone.0354822.s001.docx]

Supplementary Figure 1. Cohort selection flow diagram with associated data linkage. Abbreviations: ACCIS = Alberta Continuing Care Information System; AHCIP = Alberta Health Care Insurance Plan; ACR = Alberta Cancer Registry; COVID-19 = coronavirus disease 2019; DAD = Discharge Abstract Database; FY = fiscal year; Imm/ARI = Immunization and Adverse Reactions to Immunizations; LIS = Laboratory Information System; NACRS = National Ambulatory Care Reporting System; PC = Practitioner Claims; PHN = Personal Health Number; PIN = Pharmaceutical Information Network; REG = Population Registry; RT-PCR = reverse transcription polymerase chain reaction; SARS-CoV-2 = severe acute respiratory syndrome coronavirus 2; VS = Vital Statistics.
