# Supplementary material for: Factors associated with severe COVID-19 outcomes among adults with at least a primary vaccination schedule: A retrospective cohort study from Alberta, Canada
Source: PLoS One. 2026 Aug 17;21(8):e0354822. doi: 10.1371/journal.pone.0354822 (PMC13480603; doi:10.1371/journal.pone.0354822)
Supplement: Supplementary file 2 [file pone.0354822.s002.docx]

# Supplementary Table 1. Health conditions and their associated codes and weights included in the Charlson Comorbidity Index.

| Health condition | ICD-9-CM codes | ICD-10-CA codes | Weight |
| --- | --- | --- | --- |
| Myocardial infarction | 410, 412 | I21, I22, I25.2 | 1 |
| Congestive heart failure | 398, 402, 425, 428 | I09.9, I11.0, I13.0, I13.2, I25.5, I42.0, I42.5, I42.6, I42.7, I42.8, I42.9, I43, I50, P29.0 | 1 |
| Peripheral vascular disease | 440, 441, 443, 447, 557 | I70, I71, I73.1, I73.8, I73.9, I77.1, I79.0, I79.2, K55.1, K55.8, K55.9, Z95.8, Z95.9 | 1 |
| Cerebrovascular disease | 430, 431, 432, 433, 434, 435, 436, 437, 438 | G45, G46, I60, I61, I62, I63, I64, I65, I66, I67, I68, I69, H34.0 | 1 |
| Dementia | 290, 294, 331 | F00, F01, F02, F03, G30, F05.1, G31.1 | 1 |
| Chronic pulmonary disease | 416, 490, 491, 492, 493, 494, 495, 496, 500, 501, 502, 503, 504, 505 | J40, J41, J42, J43, J44, J45, J46, J47, J60, J61, J62, J63, J64, J65, J66, J67, I27.8, I27.9, J68.4, J70.1, J70.3 | 1 |
| Connective tissue disease | 446, 710, 714, 725 | M05, M32, M33, M34, M06, M31.5, M35.1, M35.3, M36.0 | 1 |
| Peptic ulcer disease | 531, 532, 533, 534 | K25, K26, K27, K28 | 1 |
| Mild liver disease | 070, 570, 571, 573 | B18, K73, K74, K70.0, K70.1, K70.2, K70.3, K70.9, K71.7, K71.3, K71.4, K71.5, K76.0, K76.2, K76.3, K76.4, K76.8, K76.9, Z94.4 | 1 |
| Moderate/severe liver disease | 456, 572 | K70.4, K71.1, K72.1, K72.9, K76.5, K76.6, K76.7, I85.0, I85.9, I86.4, I98.2 | 3 |
| Diabetes (without complication) | 250 | E10.0, E10.l, E10.6, E10.8, E10.9, E11.0, E11.1, E11.6, E11.8, E11.9, E12.0, E12.1, E12.6, E12.8, E12.9, E13.0, E13.1, E13.6, E13.8, E13.9, E14.0, E14.1, E14.6, E14.8, E14.9 | 1 |
| Diabetes (with complication) | 250 | E10.2, E10.3, E10.4, E10.5, E10.7, E11.2, E11.3, E11.4, E11.5, E11.7, E12.2, E12.3, E12.4, E12.5, E12.7, E13.2, E13.3, E13.4, E13.5, E13.7, E14.2, E14.3, E14.4, E14.5, E14.7 | 2 |
| Hemiplegia and paraplegia | 334, 342, 343, 344 | G81, G82, G04.1, G11.4, G80.1, G80.2, G83.0, G83.1, G83.2, G83.3, G83.4, G83.9 | 2 |
| Moderate or severe renal disease | 403, 582, 583, 585, 586, 588, V56 | N18, N19, N05.2, N05.3, N05.4, N05.5, N05.6, N05.7, N25.0, I12.0, I13.1, N03.2, N03.3, N03.4, N03.5, N03.6, N03.7, Z49.0, Z49.1, Z49.2, Z94.0, Z99.2 | 2 |
| Cancer | 140-165, 170-172, 174-176, 179-195, 200- 208, 238 | C00-C26, C30-C34, C37-C41, C43, C45-C58, C60-C76, C81-C85, C88, C90- C97 | 2 |
| Metastatic Carcinoma | 196, 197, 198, 199 | C77, C78, C79, C80 | 6 |
| HIV/AIDS | 042, 043, 044 | B20, B21, B22, B24 | 6 |

To be considered as having one of the listed diseases, a participant must have ≥1 hospitalization (associated ICD-10-CA code listed in any diagnostic field) or ≥2 physician claims (associated ICD-9-CM codes listed in any diagnostic field) of the corresponding ICD within ≤2-years. Abbreviations: HIV/AIDS = Human immunodeficiency virus/acquired immunodeficiency syndrome; ICD-9-CM = International classification of diseases, ninth revision, clinical modification; ICD-10-CA = International classification of diseases, tenth revision, Canadian enhancement
