# Supplementary material for: Factors associated with severe COVID-19 outcomes among adults with at least a primary vaccination schedule: A retrospective cohort study from Alberta, Canada
Source: PLoS One. 2026 Aug 17;21(8):e0354822. doi: 10.1371/journal.pone.0354822 (PMC13480603; doi:10.1371/journal.pone.0354822)
Supplement: Supplementary file 3 [file pone.0354822.s003.docx]

# Supplementary Table 2. Administrative data case definitions used to identify health conditions of interest.

| Health condition | Algorithm | ICD-9-CM codes | ICD-10-CA codes; other codes |
| --- | --- | --- | --- |
| Asthma | ≥1 hospitalization or ≥3 ambulatory care / claims in ≤2 years | 493 | J45 |
| Cardiovascular disease (any of the below) | |  |  |
| Atrial fibrillation | ≥1 hospitalization or ≥2 claims in ≤2 years | 427.3 | I48.0 |
| Chronic heart failure | ≥1 hospitalization or ≥2 claims in ≤2 years | 398.9, 402, 404, 425.4 –425.9, 428 | I09.9, I25.5, I42.0, I42.5–I42.9, I43, I50 |
| Coronary artery disease | ≥1 hospitalization or ≥1 ambulatory care visit or ≥1 procedure or ≥2 claims in ≤2 years | 410-413 | I20–I25; *Procedure codes:* 1.IJ.57.GQ, 1.IJ.50, 1.IL.35, 1.IJ.76 |
| Peripheral artery disease | ≥1 hospitalization or ≥1 ambulatory care visit or ≥1 claim in any years | 440.2 | I70.2 |
| Stroke | ≥1 hospitalization or emergency department visit with a stroke code in the most responsible diagnostic field OR ≥1 hospitalization, emergency department visit, or ambulatory care visit* with a stroke code in a secondary diagnostic field and a z-code in the most responsible field in any years. |  | G08, G45 (excluding subcode G45.4), H34.0, H34.1, I60, I61, I62.9, I63, I64, I67.6; Z50 (excluding subcodes Z50.2, Z50.3, Z50.4), *Z51.5 (only applies to ambulatory care), Z54.8, Z54.9 |
| Chronic kidney disease | ≥1 hospitalization or ≥3 claims in ≤1 year OR mean eGFR <60 mL/min*1.73 m^2^ or mean albuminuria >30 mg/g over 1-year | 583-586, 592, 593.9 | N00-N23; *Laboratory results:* eGFR and albuminuria |
| Chronic obstructive pulmonary disease | ≥35 years with ≥1 hospitalization or claim in any years | 491-492, 496 | J41–J44 |
| Dementia | ≥1 hospitalization or ≥2 claims in ≤2 years | 290, 294.1, 331.2 | F00–F03, F05.1, G30, G31.1 |
| Diabetes | ≥1 hospitalization or ≥2 claims in ≤2 years | 250 | E10-E14 |
| Down’s syndrome | ≥1 hospitalization, ambulatory visit, or claim in any year | 758 | Q90 |
| Dyslipidemia | ≥1 hospitalization, ambulatory care visit, claim, or lab in ≤3 years; condition present for 3 years | 272 | E78; *Laboratory* *results*: LDL ≥3.5mmol/L or non-HDL ≥4.3mmol/L or apolipoprotein B100 ≥1.2 g/L |
| Hypertension | ≥1 hospitalization or ≥2 claims in ≤2 years | 401-405 | I10-I13, I15 |
| Immunocompromised (any of the below) | | | |
| Autoimmune disorders |  |  |  |
| Inflammatory bowel disease | ≥2 hospitalizations, ≥2 ambulatory care visits, or ≥4 claims in ≤2 years | 555, 556 | K50, K51 |
| Multiple sclerosis | ≥1 hospitalization, or ≥5 claims from ambulatory care / claims in ≤2 years | 340 | G35 |
| Psoriasis | ≥1 hospitalization or ≥2 claims | 696 | L40.0-L40.4, L40.8, L40.9 |
| Psoriatic arthritis | ≥1 hospitalization or ≥3 claims for seronegative SpA (≥1 by a rheumatologist, internal medicine) and ≥1 claim for psoriasis | 696 (psoriasis), 721 (SpA) | L40.5, M07.0-M07.3, M09.0 |
| Rheumatoid arthritis | ≥1 hospitalization or ≥3 claims with ≥1 by a specialist (rheumatologist, orthopedic surgeon, internal medicine) in ≤2 years | 714 | M05, M06 |
| Systematic autoimmune rheumatoid disease | ≥1 hospitalization or ≥3 claims | 710 | M32-M34, M35.0, M35.8, M35.9, M36.0 |
| Cancer and receiving chemotherapy or an immunocompromising drug | ≥1 hospitalization or ≥2 claims in ≤2 years and received chemotherapy or an immunocompromising drug ≤1 year before index date | 140-165, 170-172, 174-176, 179-195, 196-199, 200-208, 238 | C00-C26, C30-C34, C37-C41, C43, C45-C58, C60-C76, C77-C80, C81-C85, C88, C90-C97, D01.0-D01.3, D02.2, D05-D06, D07.5; *Health services code for chemotherapy:* 13.55A; *Procedure codes for chemotherapy:* 1.ZZ.35.CA-M0, 1.ZZ.35.CA-M5, 1.ZZ.35.CA-M9, 1.ZZ.35.HA-M0, 1.ZZ.35.HA-M5, 1.ZZ.35.HA-M9, 1.ZZ.35.YA-M0, 1.ZZ.35.YA-M5, 1.ZZ.35.YA-M9; *Drug codes:* see bottom of table |
| History of solid organ or bone marrow transplant | ≥1 procedure | V42.0-V42.2, V42.4, V42.6-V42.9 | Z48.2, Z94 (not including: Z94.5, Z94.83, Z94.88, Z94.9); *Procedure codes:* 1.PC.85, 1.OA.85, 1.HY.85, 1.HZ.85, 1.GR.85, 1.OJ.85, 1.GT.85, 1.OK.85, 1.NK.85, 1.NP.85, 1.WY.19, 1.LZ.19.HH-U7, 1.LZ.19.HH-U8 |
| Receiving dialysis | ≥1 hospitalization or ambulatory care visit in ≤2 years before the index date | V45.1, V56 | Z49, Z99.2; *Health services code:* 13.99 A, B, C, D, O, or OA |
| Other immunocompromising conditions | |  |  |
| Diseases of the blood | ≥1 hospitalization |  | D70-D72, D73.0-D73.2 |
| Human immunodeficiency virus | ≥1 hospitalization or ≥3 claims in ≤3 years | 042-044 | B20-B24 |
| Immune system disorders | ≥1 hospitalization, emergency department visit , or claim OR ≥1 dispensation for an immunocompromising drug ≤1 year before index date | 279 | D80-D84, D89; *Drug codes:* see bottom of table |
| Sickle cell anemia | ≥1 hospitalization or ≥2 claims in ≤1 year | 282.6 | D57.0-D57.2, D57.8 |
| Spleen malformations or removal | ≥1 hospitalization |  | Q89.0; *Procedure codes:* 1.OB.87, 1.OB.89 |
| Treated for tuberculosis | ≥1 hospitalization or ≥1 claim | 010-018 | A15-A19 |
| A mental health disorder (any of the below) | |  |  |
| Bipolar disorder | ≥1 hospitalization OR ≥3 claims OR ≥1 claim and ≥3 dispensations for bipolar disorder treatment drugs in ≤5 years | 296 | F31; *Drug codes:* ATC N05AN01, N03AF01, N03AG01, N03AX09 |
| Depression | ≥1 hospitalization or ≥2 claims in ≤2 years | 300.4, 311 | F32, F33, F34.1 |
| Schizophrenia | ≥1 hospitalization or ≥2 claims in ≤2 years | 295 | F20, F21, F23.2, F25 |
| Obesity | ≥1 hospitalization, ambulatory visit, or claim in any years (ICD or BMI fee-modifier code in any years); excluded if no code listed after most recent dispensation for a weight-loss medication OR had bariatric surgery | 278.0, V77.8 | E66; *BMI modifier code:* BMI^^^; *Drug codes:* ATC A08AB01, A08AA62; or DIN 02437899; *Health services codes:* 55.8A, 55.8B 55.9A, or 56.93; *Procedure code:* 1.NF.78.^^ |
| Parkinson’s disease | ≥40 years with ≥1 hospitalization; OR ≥2 claims (at least 30 days between claims); OR ≥1 hospitalization / claim and ≥ 1 drug dispensation for Parkinson’s disease treatment in ≤1 year | 332 | G20, G21.0, G21.1, G21.2, G21.3, G21.4, G21.8, G21.9, G22, F02.3; *Drug codes:* ATC N04BD01, N04BD01, N04BA01, N04BA03 |
| Pregnant | Female sex with ≥1 hospitalization, ambulatory visit, or claim with ICD code for pregnancy OR delivery ≤6 months after index date (excluding abortion / miscarriage ICD codes) | 632-637, 640-648, 650-665, V22, V23, V27 | O00, O02.1, O10-O16, O20-O29, O30-O48, O60-O75, Z34, Z35, Z37; *Health services codes:* 87.0X, 87.2X; *Procedure codes:* 5.CA.88, 5.CA.89, 5.CA.90, 5.CA.93, 5.MD.5, 5.MD.60 |
| Pulmonary hypertension | ≥1 hospitalization | 416 | I27 |
| ATC codes for immunocompromising drugs: H02AA02, H02AB01, H02AB02, H02AB04, H02AB06, H02AB07, H02AB08, H02AB09, H02AB10, H02AB57, H02BX01, L01AA01, L01AA02, L01AA03, L01AA05, L01AA06, L01AA09, L01AB01, L01AB02, L01AC01, L01AD01, L01AD02, L01AD04, L01AX03, L01AX04, L01BA01, L01BA03, L01BA04, L01BA05, L01BB02, L01BB03, L01BB04, L01BB05, L01BB06, L01BB07, L01BC01, L01BC02, L01BC05, L01BC06, L01BC07, L01BC08, L01BC52, L01BC59, L01CA01, L01CA02, L01CA03, L01CA04, L01CB01, L01CB02, L01CD01, L01CD02, L01CD04, L01CE01, L01CE02, L01CX01, L01DA01, L01DB01, L01DB02, L01DB03, L01DB06, L01DB07, L01DB09, L01DC01, L01DC03, L01EA01, L01EA02, L01EA03, L01EA04, L01EA05, L01EA06, L01EB01, L01EB02, L01EB03, L01EB04, L01EB07, L01EC01, L01EC02, L01EC03, L01ED01, L01ED02, L01ED03, L01ED04, L01ED05, L01EE01, L01EE02, L01EE03, L01EE04, L01EF01, L01EF02, L01EF03, L01EG01, L01EG02, L01EH01, L01EH02, L01EH03, L01EJ01, L01EJ02, L01EK01, L01EL01, L01EL02, L01EL03, L01EM01, L01EM03, L01EN01, L01EN02, L01EN03, L01EX01, L01EX02, L01EX03, L01EX04, L01EX05, L01EX07, L01EX08, L01EX09, L01EX10, L01EX12, L01EX13, L01EX14, L01EX17, L01EX19, L01EX21, L01EX22, L01EX23, L01FA, L01FA01, L01FA02, L01FA03, L01FB01, L01FC01, L01FC02, L01FD01, L01FD02, L01FD03, L01FD04, L01FE01, L01FE02, L01FE03, L01FF01, L01FF02, L01FF03, L01FF04, L01FF05, L01FF06, L01FF07, L01FG01, L01FG02, L01FX02, L01FX03, L01FX04, L01FX05, L01FX06, L01FX07, L01FX08, L01FX09, L01FX10, L01FX12, L01FX13, L01FX14, L01FX17, L01FX18, L01X, L01XA01, L01XA02, L01XA03, L01XB01, L01XD01, L01XD03, L01XD04, L01XE, L01XF01, L01XG01, L01XG02, L01XG03, L01XH01, L01XH02, L01XJ01, L01XJ03, L01XK01, L01XK02, L01XK04, L01XL03, L01XL04, L01XL05, L01XL06, L01XL07, L01XX01, L01XX02, L01XX03, L01XX05, L01XX08, L01XX11, L01XX23, L01XX24, L01XX27, L01XX35, L01XX41, L01XX44, L01XX52, L01XX59, L01XX66, L01XX69, L01XX73, L01XX74, L01XX75, L01XY01, L01XY02, L02BA01, L02BA03, L02BB01, L02BB02, L02BB03, L02BB04, L02BB05, L02BB06, L02BG01, L02BG02, L02BG03, L02BG04, L02BG06, L02BX02, L02BX03, L03AA02, L03AA12, L03AA13, L03AB, L03AC01, L03AX03, L03AX12, L03AX13, L03AX16, L04AA02, L04AA03, L04AA04, L04AA06, L04AA10, L04AA13, L04AA15, L04AA18, L04AA21, L04AA23, L04AA24, L04AA25, L04AA26, L04AA27, L04AA29, L04AA31, L04AA32, L04AA33, L04AA34, L04AA36, L04AA37, L04AA38, L04AA40, L04AA42, L04AA43, L04AA44, L04AA47, L04AA48, L04AA50, L04AA51, L04AA52, L04AA54, L04AA56, L04AA59, L04AB01, L04AB02, L04AB04, L04AB05, L04AB06, L04AC01, L04AC02, L04AC03, L04AC05, L04AC07, L04AC08, L04AC10, L04AC11, L04AC12, L04AC13, L04AC14, L04AC16, L04AC17, L04AC18, L04AC19, L04AC21, L04AC22, L04AD01, L04AD02, L04AX01, L04AX02, L04AX03, L04AX04, L04AX06, V10XX02, X10XA53. | | | |

Case definitions were applied over a 5-year period before the index date, unless otherwise stated. Abbreviations: ATC = anatomical therapeutic chemical; BMI = body mass index; DIN = drug identification number; eGFR = estimated glomerular filtration rate; HDL = high-density lipoprotein; ICD-9-CM: International Classification of Disease – Version 9 – Clinical Modification (Alberta specific); ICD-10-CA: International Classification of Disease – Version 10 – Canadian Enhancement; LDL = low-density lipoprotein; SpA = spondyloarthropathies.
