# Supplementary material for: Factors associated with severe COVID-19 outcomes among adults with at least a primary vaccination schedule: A retrospective cohort study from Alberta, Canada
Source: PLoS One. 2026 Aug 17;21(8):e0354822. doi: 10.1371/journal.pone.0354822 (PMC13480603; doi:10.1371/journal.pone.0354822)
Supplement: Supplementary file 4 [file pone.0354822.s004.docx]

# Supplementary Table 3. ICD-10-CA codes used to define a COVID-19-related hospital admission.

| ICD-10-CA code | Description |
| --- | --- |
| *Code located within any diagnostic field:* | |
| U07.1 | COVID-19 virus identified. Assigned for COVID-19 that is confirmed by a positive COVID-19 test result, or when the physician or primary care provider or infection control staff documented a positive COVID-19 test result. |
|  |  |
| *Code located in a secondary diagnostic field:* | |
| U07.3 | Multisystem inflammatory syndrome associated with COVID-19. Assigned when the patient is diagnosed with multisystem inflammatory syndrome associated with COVID-19. |
|  |  |
| *Code located in the most responsible diagnostic field and a positive SARS-CoV-2 RT-PCR test result ≤14 days prior to or during the hospital admission:* | |
| J04 | Acute laryngitis and tracheitis |
| J09 | Influenza due to identified Avian influenza virus |
| J10 | Influenza due to other identified influenza virus |
| J11 | Influenza, virus not identified |
| J12 | Viral pneumonia, not elsewhere classified |
| J13 | Pneumonia due to Streptococcus pneumoniae |
| J14 | Pneumonia due to Haemophilus influenzae |
| J15 | Bacterial pneumonia, not elsewhere classified |
| J16 | Pneumonia due to other infectious organisms, not elsewhere classified |
| J17 | Pneumonia in diseases classified elsewhere |
| J18 | Pneumonia, organism unspecified |
| J20 | Acute bronchitis |
| J21 | Acute bronchiolitis |
| J22 | Unspecified acute lower respiratory infection |
| J80 | Adult respiratory distress syndrome |
| U04 | Severe acute respiratory syndrome [SARS] |

Abbreviations: COVID-19 = coronavirus disease 2019; ICD-10-CA = International Classification of Disease – Version 10 – Canadian Enhancement ; RT-PCR = reverse transcription polymerase chain reaction; SARS-CoV-2 = severe acute respiratory syndrome coronavirus 2.
