# Supplementary material for: Factors associated with severe COVID-19 outcomes among adults with at least a primary vaccination schedule: A retrospective cohort study from Alberta, Canada
Source: PLoS One. 2026 Aug 17;21(8):e0354822. doi: 10.1371/journal.pone.0354822 (PMC13480603; doi:10.1371/journal.pone.0354822)
Supplement: Supplementary file 5 [file pone.0354822.s005.docx]

Supplementary Table 4. Cumulative 1-year incidence and incidence density rates of severe COVID-19 outcome events among adults who completed a primary vaccination series.

|  | All severe COVID-19 outcomes | | | | |  | Type of severe COVID-19 outcome | | | | | | | | | | | | | | | | |
| --- | --- | --- | --- | --- | --- | --- | --- | --- | --- | --- | --- | --- | --- | --- | --- | --- | --- | --- | --- | --- | --- | --- | --- |
|  |  |  |  |  |  |  | Hospitalization (non-intensive care unit) | | | | |  | Intensive care unit admission | | | | |  | Death | | | | |
|  | # of events | # of events | # of incident events | Cumulative  1-year incidence | Incidence rate |  | # of events | # of events | # of incident events | Cumulative  1-year incidence | Incidence rate |  | # of events | # of events | # of incident events | Cumulative  1-year incidence | Incidence rate |  | # of events | # of events | # of incident events | Cumulative  1-year incidence | Incidence rate |
|  | (total) | (in 1 year) | (in 1 year) | (per 10,000 persons) | (per 1,000 person-years) |  | (total) | (in 1 year) | (in 1 year) | (per 10,000 persons) | (per 1,000 person-years) |  | (total) | (in 1 year) | (in 1 year) | (per 10,000 persons) | (per 1,000 person-years) |  | (total) | (in 1 year) | (in 1 year) | (per 10,000 persons) | (per 1,000 person-years) |
| Overall | 6,191 | 8,476 | 7,532 | 28.5 | 2.2 |  | 4,789 | 6,530 | 6,442 | 24.4 | 1.9 |  | 432 | 550 | 550 | 2.1 | 0.2 |  | 970 | 1,396 | 1,396 | 5.3 | 0.4 |
|  |  |  |  |  |  |  |  |  |  |  |  |  |  |  |  |  |  |  |  |  |  |  |  |
| **Demographic characteristics** | | | |  |  |  |  |  |  |  |  |  |  |  |  |  |  |  |  |  |  |  |  |
| Age category (years) |  |  |  |  |  |  |  |  |  |  |  |  |  |  |  |  |  |  |  |  |  |  |  |
| 18-39 | 933 | 943 | 928 | 10.1 | 0.9 |  | 862 | 877 | 870 | 9.4 | 0.8 |  | 48 | 46 | 46 | 0.5 | 0.0 |  | 23 | 20 | 20 | 0.2 | 0.0 |
| 40-49 | 4492 | 517 | 485 | 10.1 | 0.9 |  | 366 | 414 | 405 | 8.4 | 0.7 |  | 58 | 71 | 71 | 1.5 | 0.1 |  | 25 | 32 | 32 | 0.7 | 0.1 |
| 50-59 | 733 | 898 | 825 | 18.1 | 1.6 |  | 578 | 714 | 698 | 15.3 | 1.4 |  | 81 | 98 | 98 | 2.2 | 0.2 |  | 74 | 86 | 86 | 1.9 | 0.2 |
| 60-64 | 534 | 671 | 604 | 26.5 | 2.8 |  | 400 | 503 | 493 | 21.6 | 2.3 |  | 68 | 82 | 82 | 3.6 | 0.4 |  | 66 | 86 | 86 | 3.8 | 0.4 |
| ≥65 | 3,542 | 5,447 | 4,690 | 84.7 | 8.1 |  | 2,583 | 4,022 | 3,976 | 71.8 | 6.8 |  | 177 | 253 | 253 | 4.6 | 0.5 |  | 782 | 1,172 | 1,172 | 21.2 | 2.1 |
| Sex |  |  |  |  |  |  |  |  |  |  |  |  |  |  |  |  |  |  |  |  |  |  |  |
| Female | 2,891 | 3,924 | 3,545 | 26.4 | 2.1 |  | 2,334 | 3,108 | 3,064 | 22.8 | 1.8 |  | 159 | 201 | 201 | 1.5 | 0.1 |  | 398 | 615 | 615 | 4.6 | 0.3 |
| Male | 3,300 | 4,552 | 3,987 | 30.8 | 2.3 |  | 2,455 | 3,422 | 3,378 | 26.1 | 1.9 |  | 273 | 349 | 349 | 2.7 | 0.2 |  | 572 | 781 | 781 | 6.0 | 0.5 |
| Other | 0 | 0 | 0 | 0.0 | 0.0 |  | 0 | 0 | 0 | 0.0 | 0.0 |  | 0 | 0 | 0 | 0.0 | 0.0 |  | 0 | 0 | 0 | 0.0 | 0.0 |
| Residence |  |  |  |  |  |  |  |  |  |  |  |  |  |  |  |  |  |  |  |  |  |  |  |
| Rural/urban |  |  |  |  |  |  |  |  |  |  |  |  |  |  |  |  |  |  |  |  |  |  |  |
| Rural | 1,132 | 1,431 | 1,265 | 40.7 | 3.2 |  | 841 | 1,057 | 1,049 | 33.8 | 2.6 |  | 107 | 125 | 125 | 4.0 | 0.3 |  | 184 | 249 | 249 | 8.0 | 0.6 |
| Urban | 5,059 | 7,045 | 6,267 | 26.9 | 2.1 |  | 3,948 | 5,473 | 5,393 | 23.2 | 1.8 |  | 325 | 425 | 425 | 1.8 | 0.1 |  | 786 | 1,147 | 1,147 | 4.9 | 0.4 |
| Long-term care |  |  |  |  |  |  |  |  |  |  |  |  |  |  |  |  |  |  |  |  |  |  |  |
| Yes | 143 | 271 | 241 | 175.8 | 16.2 |  | 49 | 94 | 92 | 67.1 | 6.1 |  | <10 | <10 | <10 | NA | NA |  | 92 | 172 | 172 | 125.4 | 11.7 |
| No | 6,048 | 8,205 | 7,291 | 27.8 | 2.1 |  | 4,740 | 6,436 | 6,350 | 24.2 | 1.9 |  | 423-431 | 541-549 | 541-549 | 2.1 | 0.2 |  | 878 | 1,224 | 1,224 | 4.7 | 0.4 |
|  |  |  |  |  |  |  |  |  |  |  |  |  |  |  |  |  |  |  |  |  |  |  |  |
| **Socioeconomic status** |  |  |  |  |  |  |  |  |  |  |  |  |  |  |  |  |  |  |  |  |  |  |  |
| Material deprivation index |  |  |  |  |  |  |  |  |  |  |  |  |  |  |  |  |  |  |  |  |  |  |  |
| 1 (most well off) | 799 | 1,209 | 1,077 | 21.3 | 1.6 |  | 633 | 930 | 915 | 18.1 | 1.4 |  | 44 | 64 | 64 | 1.3 | 0.1 |  | 122 | 215 | 215 | 4.2 | 0.3 |
| 2 | 802 | 1,200 | 1,079 | 21.6 | 1.6 |  | 605 | 926 | 919 | 18.4 | 1.3 |  | 69 | 82 | 82 | 1.6 | 0.2 |  | 128 | 192 | 192 | 3.8 | 0.3 |
| 3 | 1,051 | 1,449 | 1,287 | 25.9 | 2.0 |  | 806 | 1,114 | 1,105 | 22.3 | 1.7 |  | 72 | 97 | 97 | 2.0 | 0.2 |  | 173 | 238 | 238 | 4.8 | 0.4 |
| 4 | 1,254 | 1,713 | 1,519 | 29.5 | 2.2 |  | 961 | 1,332 | 1,308 | 25.4 | 1.9 |  | 81 | 103 | 103 | 2.0 | 0.2 |  | 212 | 278 | 278 | 5.4 | 0.4 |
| 5 (most deprived) | 1,776 | 2,098 | 1,860 | 37.5 | 3.1 |  | 1,374 | 1,605 | 1,584 | 32.0 | 2.6 |  | 143 | 165 | 165 | 3.3 | 0.3 |  | 259 | 328 | 328 | 6.6 | 0.5 |
| Missing | 509 | 807 | 710 | 55.8 | 3.9 |  | 410 | 623 | 611 | 48.1 | 3.5 |  | 23 | 39 | 39 | 3.1 | 0.2 |  | 76 | 145 | 145 | 11.4 | 0.7 |
|  |  |  |  |  |  |  |  |  |  |  |  |  |  |  |  |  |  |  |  |  |  |  |  |
| **Clinical characteristics** |  |  |  |  |  |  |  |  |  |  |  |  |  |  |  |  |  |  |  |  |  |  |  |
| Charlson Comorbidity Index |  |  |  |  |  |  |  |  |  |  |  |  |  |  |  |  |  |  |  |  |  |  |  |
| Category; burden |  |  |  |  |  |  |  |  |  |  |  |  |  |  |  |  |  |  |  |  |  |  |  |
| 0; none | 1,820 | 2,053 | 1,939 | 10.2 | 0.9 |  | 1,539 | 1,743 | 1,727 | 9.1 | 0.8 |  | 124 | 146 | 146 | 0.8 | 0.1 |  | 157 | 164 | 164 | 0.9 | 0.1 |
| 1-2; mild | 2,229 | 3,105 | 2,773 | 47.6 | 4.1 |  | 1,717 | 2,368 | 2,340 | 40.2 | 3.5 |  | 168 | 206 | 206 | 3.5 | 0.3 |  | 344 | 531 | 531 | 9.1 | 0.7 |
| 3-4; moderate | 1,169 | 1,805 | 1,555 | 151.5 | 13.8 |  | 841 | 1,326 | 1,304 | 127.1 | 11.5 |  | 84 | 113 | 113 | 11.0 | 1.2 |  | 244 | 366 | 366 | 35.7 | 3.4 |
| ≥5; severe | 973 | 1,513 | 1,265 | 267.2 | 24.9 |  | 692 | 1,093 | 1,071 | 226.2 | 21.0 |  | 56 | 85 | 85 | 18.0 | 1.7 |  | 225 | 335 | 335 | 70.8 | 7.0 |
| Health conditions of interest |  |  |  |  |  |  |  |  |  |  |  |  |  |  |  |  |  |  |  |  |  |  |  |
| Dialysis |  |  |  |  |  |  |  |  |  |  |  |  |  |  |  |  |  |  |  |  |  |  |  |
| Yes | 97 | 200 | 173 | 583.1 | 52.4 |  | 69 | 144 | 140 | 471.9 | 42.4 |  | 11 | 22 | 22 | 74.1 | 7.1 |  | 17 | 34 | 34 | 114.6 | 11.0 |
| No | 6,094 | 8,276 | 7,359 | 27.9 | 2.2 |  | 4,720 | 6,386 | 6,302 | 23.9 | 1.9 |  | 421 | 528 | 528 | 2.0 | 0.2 |  | 953 | 1,362 | 1,362 | 5.2 | 0.4 |
| Pulmonary hypertension |  |  |  |  |  |  |  |  |  |  |  |  |  |  |  |  |  |  |  |  |  |  |  |
| Yes | 61 | 97 | 83 | 360.2 | 33.5 |  | 46 | 71 | 69 | 299.5 | 28.3 |  | <10 | <10 | <10 | NA | NA |  | 10 | 17 | 17 | 73.8 | 6.4 |
| No | 6,130 | 8,379 | 7,449 | 28.2 | 2.2 |  | 4,743 | 6,459 | 6,373 | 24.2 | 1.9 |  | 423-431 | 423-431 | 423-431 | 2.1 | 0.2 |  | 960 | 1,379 | 1,379 | 5.2 | 0.4 |
| Dementia |  |  |  |  |  |  |  |  |  |  |  |  |  |  |  |  |  |  |  |  |  |  |  |
| Yes | 694 | 1,151 | 1,007 | 246.3 | 23.5 |  | 457 | 746 | 742 | 181.5 | 17.5 |  | 16 | 22 | 22 | 5.4 | 0.6 |  | 221 | 383 | 383 | 93.7 | 8.5 |
| No | 5,497 | 7,325 | 6,525 | 25.1 | 2.0 |  | 4,332 | 5,784 | 5,700 | 21.9 | 1.7 |  | 416 | 528 | 528 | 2.0 | 0.2 |  | 749 | 1,013 | 1,013 | 3.9 | 0.3 |
| History of a transplant |  |  |  |  |  |  |  |  |  |  |  |  |  |  |  |  |  |  |  |  |  |  |  |
| Yes | 163 | 392 | 323 | 294.0 | 18.7 |  | 111 | 274 | 261 | 237.6 | 15.2 |  | 26 | 57 | 57 | 51.9 | 3.7 |  | 26 | 61 | 61 | 55.5 | 3.7 |
| No | 6,028 | 8,084 | 7,209 | 27.4 | 2.1 |  | 4,678 | 6,256 | 6,181 | 23.5 | 1.8 |  | 406 | 493 | 493 | 1.9 | 0.2 |  | 944 | 1,335 | 1,335 | 5.1 | 0.4 |
| Parkinson's disease |  |  |  |  |  |  |  |  |  |  |  |  |  |  |  |  |  |  |  |  |  |  |  |
| Yes | 106 | 179 | 156 | 202.4 | 18.2 |  | 79 | 129 | 129 | 167.3 | 15.4 |  | <10 | <10 | <10 | NA | NA |  | 24 | 47 | 47 | 61.0 | 4.7 |
| No | 6,085 | 8,297 | 7,376 | 28.0 | 2.2 |  | 4,710 | 6,401 | 6,313 | 24.0 | 1.9 |  | 423-431 | 541-549 | 541-549 | 2.1 | 0.2 |  | 946 | 1,349 | 1,349 | 5.1 | 0.4 |
| Cancer |  |  |  |  |  |  |  |  |  |  |  |  |  |  |  |  |  |  |  |  |  |  |  |
| Yes | 482 | 840 | 694 | 166.6 | 14.1 |  | 332 | 610 | 589 | 141.4 | 11.7 |  | 38 | 59 | 59 | 14.2 | 1.4 |  | 112 | 171 | 171 | 41.0 | 4.1 |
| No | 5,785 | 7,636 | 6,838 | 26.3 | 2.1 |  | 4,457 | 5,920 | 5,853 | 22.5 | 1.8 |  | 394 | 491 | 491 | 1.9 | 0.2 |  | 858 | 1,225 | 1,225 | 4.7 | 0.3 |
| Chronic obstructive pulmonary disease | | | |  |  |  |  |  |  |  |  |  |  |  |  |  |  |  |  |  |  |  |  |
| Yes | 1,535 | 2,193 | 1,871 | 147.2 | 13.4 |  | 1,119 | 1,627 | 1,595 | 125.5 | 11.3 |  | 95 | 123 | 123 | 9.7 | 1.0 |  | 321 | 443 | 443 | 34.9 | 3.3 |
| No | 4,656 | 6,283 | 5,661 | 22.5 | 1.7 |  | 3,670 | 4,903 | 4,847 | 19.3 | 1.5 |  | 337 | 427 | 427 | 1.7 | 0.1 |  | 649 | 953 | 953 | 3.8 | 0.3 |
| Chronic kidney disease |  |  |  |  |  |  |  |  |  |  |  |  |  |  |  |  |  |  |  |  |  |  |  |
| Yes | 1,655 | 2,531 | 2,159 | 147.4 | 13.2 |  | 1,198 | 1,863 | 1,827 | 124.7 | 11.1 |  | 112 | 159 | 159 | 10.9 | 1.1 |  | 345 | 509 | 509 | 34.7 | 3.3 |
| No | 4,536 | 5,945 | 5,373 | 21.6 | 1.7 |  | 3,591 | 4,667 | 4,615 | 18.5 | 1.5 |  | 320 | 391 | 391 | 1.6 | 0.1 |  | 625 | 887 | 887 | 3.6 | 0.3 |
| Down's syndrome |  |  |  |  |  |  |  |  |  |  |  |  |  |  |  |  |  |  |  |  |  |  |  |
| Yes | 12 | 18 | 16 | 147.7 | 12.6 |  | <10 | 14 | 14 | 129.3 | 10.3 |  | <10 | <10 | <10 | NA | NA |  | <10 | <10 | <10 | NA | NA |
| No | 6,179 | 8,458 | 7,516 | 28.5 | 2.2 |  | 4,780-4,788 | 6,516 | 6,428 | 24.4 | 1.9 |  | 423-431 | 541-549 | 541-549 | 2.1 | 0.2 |  | 961-969 | 1,387-1,395 | 1,387-1,395 | 5.3 | 0.4 |
| Cardiovascular disease |  |  |  |  |  |  |  |  |  |  |  |  |  |  |  |  |  |  |  |  |  |  |  |
| Yes | 2,549 | 3,841 | 3,313 | 119.3 | 9.7 |  | 1,871 | 2,842 | 2,795 | 100.7 | 8.1 |  | 178 | 240 | 240 | 8.6 | 0.8 |  | 500 | 759 | 759 | 27.3 | 2.2 |
| No | 3,642 | 4,635 | 4,219 | 17.9 | 1.5 |  | 2,918 | 3,688 | 3,647 | 15.4 | 1.3 |  | 254 | 310 | 310 | 1.3 | 0.1 |  | 470 | 637 | 637 | 2.7 | 0.2 |
| Diabetes |  |  |  |  |  |  |  |  |  |  |  |  |  |  |  |  |  |  |  |  |  |  |  |
| Yes | 1,991 | 2,867 | 2,502 | 91.3 | 7.9 |  | 1,481 | 2,167 | 2,136 | 77.9 | 6.7 |  | 170 | 210 | 210 | 7.7 | 0.8 |  | 340 | 490 | 490 | 17.9 | 1.6 |
| No | 4,200 | 5,609 | 5,030 | 21.3 | 1.6 |  | 3,308 | 4,363 | 4,306 | 18.2 | 1.4 |  | 262 | 340 | 340 | 1.4 | 0.1 |  | 630 | 906 | 906 | 3.8 | 0.3 |
| Pregnancy |  |  |  |  |  |  |  |  |  |  |  |  |  |  |  |  |  |  |  |  |  |  |  |
| Yes | 50 | 55 | 55 | 88.2 | 7.6 |  | 50 | 55 | 55 | 88.2 | 7.6 |  | 0 | 0 | 0 | 0.0 | 0.0 |  | 0 | 0 | 0 | 0.0 | 0.0 |
| No | 6,141 | 8,421 | 7,477 | 28.4 | 2.2 |  | 4,739 | 6,475 | 6,387 | 24.3 | 1.9 |  | 432 | 550 | 550 | 2.1 | 0.2 |  | 970 | 1,396 | 1,396 | 5.3 | 0.4 |
| Other immunocompromising conditions | | | |  |  |  |  |  |  |  |  |  |  |  |  |  |  |  |  |  |  |  |  |
| Yes | 1,443 | 2,374 | 2,029 | 94.1 | 6.9 |  | 1,046 | 1,738 | 1,697 | 78.7 | 5.7 |  | 131 | 209 | 209 | 9.7 | 0.7 |  | 266 | 427 | 427 | 19.8 | 1.5 |
| No | 4,748 | 6,102 | 5,503 | 22.7 | 1.8 |  | 3,743 | 4,792 | 4,745 | 19.6 | 1.6 |  | 301 | 341 | 341 | 1.4 | 0.1 |  | 704 | 969 | 969 | 4.0 | 0.3 |
| Immunocompromised (overall) | | |  |  |  |  |  |  |  |  |  |  |  |  |  |  |  |  |  |  |  |  |  |
| Yes | 1,692 | 2,764 | 2,571 | 88.2 | 6.5 |  | 1,243 | 2,039 | 2,160 | 74.1 | 5.5 |  | 143 | 229 | 240 | 8.5 | 0.6 |  | 306 | 496 | 547 | 18.5 | 1.4 |
| No | 4,499 | 5,712 | 4,961 | 21.8 | 1.8 |  | 3,546 | 4,491 | 4,282 | 18.8 | 1.5 |  | 289 | 321 | 310 | 1.4 | 0.1 |  | 664 | 900 | 849 | 3.8 | 0.3 |
| Hypertension |  |  |  |  |  |  |  |  |  |  |  |  |  |  |  |  |  |  |  |  |  |  |  |
| Yes | 3,416 | 5,130 | 4,458 | 72.8 | 6.2 |  | 2,536 | 3,824 | 3,767 | 61.5 | 5.3 |  | 234 | 318 | 318 | 5.2 | 0.5 |  | 646 | 988 | 988 | 16.1 | 1.4 |
| No | 2,775 | 3,346 | 3,074 | 15.2 | 1.3 |  | 2,253 | 2,706 | 2,675 | 13.2 | 1.1 |  | 198 | 232 | 232 | 1.1 | 0.1 |  | 324 | 408 | 408 | 2.0 | 0.2 |
| Autoimmune disorders |  |  |  |  |  |  |  |  |  |  |  |  |  |  |  |  |  |  |  |  |  |  |  |
| Yes | 406 | 688 | 602 | 67.4 | 5.0 |  | 305 | 531 | 519 | 58.1 | 4.1 |  | 39 | 54 | 54 | 6.0 | 0.5 |  | 62 | 103 | 103 | 11.5 | 0.9 |
| No | 5,785 | 7,788 | 6,930 | 27.2 | 2.1 |  | 4,484 | 5,999 | 5,923 | 23.2 | 1.8 |  | 393 | 496 | 496 | 1.9 | 0.2 |  | 908 | 1,293 | 1,293 | 5.1 | 0.4 |
| Obesity |  |  |  |  |  |  |  |  |  |  |  |  |  |  |  |  |  |  |  |  |  |  |  |
| Yes | 1,054 | 1,398 | 1,247 | 54.6 | 4.6 |  | 800 | 1,063 | 1,051 | 46.0 | 3.8 |  | 105 | 130 | 130 | 5.7 | 0.5 |  | 149 | 205 | 205 | 9.0 | 0.7 |
| No | 5,137 | 7,078 | 6,285 | 26.1 | 2.0 |  | 3,989 | 5,467 | 5,391 | 22.4 | 1.7 |  | 327 | 420 | 420 | 1.7 | 0.1 |  | 821 | 1,191 | 1,191 | 4.9 | 0.4 |
| Mental health disorder | | |  |  |  |  |  |  |  |  |  |  |  |  |  |  |  |  |  |  |  |  |  |
| Yes | 1,705 | 2,261 | 2,042 | 50.0 | 4.0 |  | 1,357 | 1,754 | 1,731 | 42.3 | 3.4 |  | 123 | 155 | 155 | 3.8 | 0.3 |  | 225 | 352 | 352 | 8.6 | 0.6 |
| No | 4,486 | 6,215 | 5,490 | 24.6 | 1.9 |  | 3,432 | 4,776 | 4,711 | 21.1 | 1.6 |  | 309 | 395 | 395 | 1.8 | 0.1 |  | 745 | 1,044 | 1,044 | 4.7 | 0.4 |
| Asthma |  |  |  |  |  |  |  |  |  |  |  |  |  |  |  |  |  |  |  |  |  |  |  |
| Yes | 377 | 558 | 501 | 50.2 | 3.8 |  | 314 | 454 | 443 | 44.3 | 3.4 |  | 25 | 36 | 36 | 3.6 | 0.3 |  | 38 | 68 | 68 | 6.8 | 0.4 |
| No | 5,814 | 7,918 | 7,031 | 27.7 | 2.1 |  | 4,475 | 6,076 | 5,999 | 23.6 | 1.8 |  | 407 | 514 | 514 | 2.0 | 0.2 |  | 932 | 1,328 | 1,328 | 5.2 | 0.4 |
| Dyslipidemia |  |  |  |  |  |  |  |  |  |  |  |  |  |  |  |  |  |  |  |  |  |  |  |
| Yes | 1,596 | 2,268 | 1,998 | 30.9 | 2.6 |  | 1,219 | 1,751 | 1,718 | 26.6 | 2.2 |  | 136 | 166 | 166 | 2.6 | 0.3 |  | 241 | 351 | 351 | 5.4 | 0.4 |
| No | 4,595 | 6,208 | 5,534 | 27.8 | 2.1 |  | 3,570 | 4,779 | 4,724 | 23.7 | 1.8 |  | 296 | 384 | 384 | 1.9 | 0.2 |  | 729 | 1,045 | 1,045 | 5.2 | 0.4 |
|  |  |  |  |  |  |  |  |  |  |  |  |  |  |  |  |  |  |  |  |  |  |  |  |
| **Other factors** |  |  |  |  |  |  |  |  |  |  |  |  |  |  |  |  |  |  |  |  |  |  |  |
| Prior SARS-CoV-2 infection |  |  |  |  |  |  |  |  |  |  |  |  |  |  |  |  |  |  |  |  |  |  |  |
| Yes | 226 | 249 | 237 | 16.5 | 1.3 |  | 177 | 196 | 193 | 13.5 | 1.1 |  | 16 | 13 | 13 | 0.9 | 0.1 |  | 33 | 40 | 40 | 2.8 | 0.2 |
| No | 5,965 | 8,227 | 7,295 | 29.2 | 2.3 |  | 4,612 | 6,334 | 6,249 | 25.0 | 1.9 |  | 416 | 537 | 537 | 2.2 | 0.2 |  | 937 | 1,356 | 1,356 | 5.4 | 0.4 |
| Received nirmatrelvir/ritonavir | |  |  |  |  |  |  |  |  |  |  |  |  |  |  |  |  |  |  |  |  |  |  |
| Yes | 118 | 200 | 186 | 91.5 | 8.9 |  | 100 | 173 | 167 | 82.1 | 7.8 |  | <10 | 13 | 13 | 6.4 | 0.7 |  | <10 | 14 | 14 | 6.9 | 0.7 |
| No | 6,073 | 8,276 | 7,346 | 28.0 | 2.2 |  | 4,689 | 6,357 | 6,275 | 24.0 | 1.9 |  | 423-431 | 537 | 537 | 2.1 | 0.2 |  | 961-969 | 1,382 | 1,382 | 5.3 | 0.4 |
| Early vaccination |  |  |  |  |  |  |  |  |  |  |  |  |  |  |  |  |  |  |  |  |  |  |  |
| Yes | 5,629 | 7,952 | 7,054 | 28.7 | 2.2 |  | 4,351 | 6,119 | 6,037 | 24.6 | 1.9 |  | 385 | 504 | 504 | 2.1 | 0.2 |  | 893 | 1,329 | 1,329 | 5.4 | 0.4 |
| No | 562 | 524 | 478 | 25.7 | 2.2 |  | 438 | 411 | 405 | 21.8 | 1.9 |  | 47 | 46 | 46 | 2.5 | 0.2 |  | 77 | 67 | 67 | 3.6 | 0.3 |
| Pandemic wave vaccine dose received* | | | |  |  |  |  |  |  |  |  |  |  |  |  |  |  |  |  |  |  |  |  |
| Wave 2 | 55 | 91 | 80 | 51.8 | 3.6 |  | 20 | 37 | 37 | 24.0 | 1.5 |  | <10 | <10 | <10 | NA | NA |  | 34 | 52 | 52 | 33.7 | 2.6 |
| Wave 3 (Alpha) | 3,332 | 5,737 | 5,019 | 33.4 | 2.6 |  | 2,472 | 4,316 | 4,263 | 28.3 | 2.2 |  | 227-235 | 355-363 | 355-363 | 2.4 | 0.2 |  | 625 | 1,059 | 1,059 | 7.0 | 0.6 |
| Wave 4 (Delta) | 2,418 | 2,281 | 2,096 | 21.0 | 1.8 |  | 1,993 | 1,887 | 1,855 | 18.6 | 1.6 |  | 166 | 158 | 158 | 1.6 | 0.1 |  | 259 | 236 | 236 | 2.4 | 0.2 |
| Wave 5 (Omicron) | 386 | 367 | 337 | 27.8 | 2.5 |  | 304 | 290 | 287 | 23.7 | 2.2 |  | 30 | 28 | 28 | 2.3 | 0.2 |  | 52 | 49 | 49 | 4.0 | 0.4 |

*Pandemic wave was measured on the day follow-up commenced. Abbreviations: COVID-19 = coronavirus disease 2019; NA = not applicable; SARS-CoV-2 = severe acute respiratory syndrome coronavirus 2.
