# Supplementary material for: Factors associated with severe COVID-19 outcomes among adults with at least a primary vaccination schedule: A retrospective cohort study from Alberta, Canada
Source: PLoS One. 2026 Aug 17;21(8):e0354822. doi: 10.1371/journal.pone.0354822 (PMC13480603; doi:10.1371/journal.pone.0354822)
Supplement: Supplementary file 6 [file pone.0354822.s006.docx]

# Supplementary Table 5. Expected number of adults with a severe COVID-19 outcome (per 10,000 persons) after the index date among those who completed a primary vaccination series.

|  | Any severe COVID-19 outcome | | |  | Type of severe COVID-19 outcome | | | | | | | | | | | | | | |  |
| --- | --- | --- | --- | --- | --- | --- | --- | --- | --- | --- | --- | --- | --- | --- | --- | --- | --- | --- | --- | --- |
|  |  |  |  |  | Hospitalization (non-intensive care unit) | | |  | | Intensive care unit admission | | | |  | | Death | | | |  |
|  | at 6-months | at 12-months | at 24-months |  | at 6-months | at 12-months | at 24-months | |  | | at 6-months | at 12-months | at 24-months | |  | | at 6-months | at 12-months | at 24-months | |
|  | number  [95% CI] | number  [95% CI] | number  [95% CI] |  | number  [95% CI] | number  [95% CI] | number  [95% CI] | |  | | number  [95% CI] | number  [95% CI] | number  [95% CI] | |  | | number  [95% CI] | number  [95% CI] | number  [95% CI] | |
| **Demographic characteristics** | |  |  |  |  |  |  | |  | |  |  |  | |  | |  |  |  | |
| Age category (years) | |  |  |  |  |  |  | |  | |  |  |  | |  | |  |  |  | |
| 18-39 | 3.6  [3.2-4.1] | 10.2  [9.4-11.0] | 14.7  [12.6-16.8] |  | 3.4  [3.0-3.8] | 9.6  [8.8-10.3] | 13.8  [11.7-15.9] | |  | | 0.2  [0.1-0.3] | 0.5  [0.3-0.7] | 0.8  [0.5-1.0] | |  | | 0.1  [0.0-0.1] | 0.2  [0.1-0.4] | 0.4  [0.2-0.5] | |
| 40-49 | 3.8  [3.2-4.4] | 10.3  [9.1-11.4] | 13.8  [12.3-15.3] |  | 3.2  [2.7-3.8] | 8.6  [7.5-9.6] | 11.6  [10.3-13.0] | |  | | 0.5  [0.3-0.7] | 1.5  [1.1-2.0] | 2.1  [1.4-2.8] | |  | | 0.3  [0.1-0.4] | 0.6  [0.3-0.9] | 0.8  [0.5-1.2] | |
| 50-59 | 5.4  [4.6-6.1] | 19.7  [17.9-21.6] | 32.3  [26.7-37.8] |  | 4.5  [3.8-5.2] | 16.9  [15.1-18.6] | 25.3  [22.6-28.1] | |  | | 0.6  [0.4-0.9] | 2.2  [1.6-2.8] | 5.9  [1.1-10.6] | |  | | 0.5  [0.3-0.7] | 1.8  [1.2-2.3] | 3.3  [2.5-4.2] | |
| 60-64 | 7.4  [6.1-8.6] | 37.5  [33.2-41.9] | 64.2  [50.3-78.1] |  | 6.0  [4.8-7.1] | 31.1  [27.2-35.1] | 53.5  [39.8-67.1] | |  | | 1.0  [0.6-1.5] | 4.7  [3.2-6.2] | 8.3  [6.0-10.6] | |  | | 0.8  [0.4-1.2] | 5.3  [3.6-7.0] | 8.5  [6.1-10.8] | |
| ≥65 | 20.6  [19.3-21.8] | 126.6  [120.0-133.2] | 243.6  [227.7-259.4] |  | 17.0  [15.8-18.2] | 109.9  [103.7-116.1] | 207.1  [192.4-221.7] | |  | | 1.6  [1.3-2.0] | 6.5  [5.1-7.8] | 11.6  [9.3-13.9] | |  | | 5.6  [4.9-6.3] | 32.8  [29.4-36.1] | 65.2  [57.9-72.6] | |
| Sex |  |  |  |  |  |  |  | |  | |  |  |  | |  | |  |  |  | |
| Female | 6.8  [6.3-7.3] | 24.5  [23.3-25.7] | 43.4  [39.6-47.2] |  | 5.9  [5.4-6.3] | 21.7  [20.6-22.8] | 37.3  [33.9-40.6] | |  | | 0.5  [0.3-0.6] | 1.3  [1.0-1.6] | 2.8  [1.8-3.9] | |  | | 1.0  [0.8-1.2] | 3.6  [3.1-4.0] | 7.1  [5.5-8.7] | |
| Male | 8.6  [8.1-9.2] | 26.6  [25.4-27.8] | 46.8  [39.5-54.1] |  | 7.2  [6.7-7.7] | 22.6  [21.5-23.7] | 38.8  [32.4-45.3] | |  | | 0.9  [0.8-1.1] | 2.6  [2.2-3.0] | 3.6  [3.1-4.1] | |  | | 1.7  [1.5-1.9] | 5.1  [4.6-5.7] | 10.3  [6.9-13.6] | |
| Residence |  |  |  |  |  |  |  | |  | |  |  |  | |  | |  |  |  | |
| Rural | 12.3  [11.0-13.6] | 37.1  [34.3-39.9] | 58.6  [52.6-64.7] |  | 10.1  [8.9-11.3] | 30.7  [28.2-33.3] | 46.2  [42.6-49.9] | |  | | 1.5  [1.0-1.9] | 4.1  [3.1-5.0] | 7.0  [4.2-9.8] | |  | | 2.3  [1.7-2.9] | 7.4  [6.2-8.7] | 12.2  [8.0-16.3] | |
| Urban | 7.1  [6.8-7.5] | 23.9  [23.0-24.7] | 42.7  [38.7-46.7] |  | 6.1  [5.7-6.4] | 20.9  [20.1-21.7] | 36.9  [33.1-40.6] | |  | | 0.6  [0.5-0.7] | 1.7  [1.4-1.9] | 2.6  [2.2-2.9] | |  | | 1.2  [1.1-1.4] | 3.9  [3.6-4.3] | 7.9  [6.3-9.4] | |
| Long-term care/supportive living | | | |  |  |  |  | |  | |  |  |  | |  | |  |  |  | |
| Yes | 43.7  [31.1-56.3] | 317.3  [196.4-436.7] | 1150.8  [788.8-1498.7] |  | 20.2  [11.5-28.8] | 92.3  [26.2-157.9] | 345.7  [153.1-534.4] | |  | | 0.0  [0.0-0.0] | 21.0  [0.0-62.1] | 21.0  [0.0-62.1] | |  | | 31.5  [20.8-42.3] | 239.6  [136.8-341.3] | 924.2  [589.4-1247.1] | |
| No | 7.6  [7.2-7.9] | 25.1  [24.2-25.9] | 43.0  [39.8-46.2] |  | 6.5  [6.1-6.8] | 22.0  [21.2-22.8] | 37.2  [34.3-40.2] | |  | | 0.7  [0.6-0.8] | 1.9  [1.7-2.2] | 3.3  [2.6-4.0] | |  | | 1.2  [1.1-1.4] | 4.0  [3.6-4.3] | 7.2  [6.1-8.4] | |
|  |  |  |  |  |  |  |  | |  | |  |  |  | |  | |  |  |  | |
| **Socioeconomic status** | |  |  |  |  |  |  | |  | |  |  |  | |  | |  |  |  | |
| Material Deprivation | |  |  |  |  |  |  | |  | |  |  |  | |  | |  |  |  | |
| 1 | 5.7  [5.0-6.4] | 20.0  [18.3-21.8] | 39.0  [29.7-48.3] |  | 4.9  [4.3-5.6] | 17.5  [15.8-19.2] | 33.1  [24.2-41.9] | |  | | 0.4  [0.2-0.6] | 1.1  [0.7-1.5] | 1.7  [1.2-2.3] | |  | | 1.0  [0.7-1.3] | 3.2  [2.5-3.9] | 7.2  [4.2-10.3] | |
| 2 | 5.9  [5.2-6.6] | 20.3  [18.6-22.1] | 31.6  [26.6-36.5] |  | 4.9  [4.3-5.5] | 17.0  [15.4-18.6] | 26.9  [22.0-31.8] | |  | | 0.6  [0.4-0.8] | 2.0  [1.5-2.5] | 2.5  [1.9-3.2] | |  | | 1.0  [0.7-1.3] | 3.5  [2.8-4.2] | 5.6  [4.1-7.1] | |
| 3 | 7.1  [6.3-7.9] | 23.6  [21.8-25.4] | 46.2  [36.3-56.0] |  | 6.1  [5.4-6.8] | 20.6  [18.9-22.3] | 39.1  [30.8-47.5] | |  | | 0.7  [0.4-0.9] | 1.8  [1.3-2.3] | 2.5  [1.8-3.1] | |  | | 1.3  [1.0-1.7] | 4.3  [3.5-5.0] | 9.4  [4.1-14.6] | |
| 4 | 7.7  [6.9-8.5] | 25.7  [23.9-27.6] | 44.2  [37.0-51.4] |  | 6.2  [5.5-6.9] | 22.6  [20.9-24.3] | 36.4  [30.5-42.3] | |  | | 0.8  [0.6-1.1] | 1.5  [1.1-1.9] | 2.8  [2.1-3.6] | |  | | 1.6  [1.2-1.9] | 4.6  [3.8-5.3] | 9.8  [5.7-13.9] | |
| 5 | 12.1  [11.1-13.0] | 36.0  [34.0-38.1] | 58.2  [52.2-64.1] |  | 10.5  [9.6-11.4] | 31.2  [29.3-33.2] | 49.7  [44.3-55.1] | |  | | 1.0  [0.7-1.3] | 3.2  [2.5-3.8] | 6.0  [3.6-8.4] | |  | | 1.9  [1.5-2.3] | 5.8  [5.0-6.7] | 10.0  [8.4-11.6] | |
|  |  |  |  |  |  |  |  | |  | |  |  |  | |  | |  |  |  | |
| **Clinical characteristics** | | |  |  |  |  |  | |  | |  |  |  | |  | |  |  |  | |
| Charlson Comorbidity Index Category; burden | | | | | |  |  | |  | |  |  |  | |  | |  |  |  | |
| 0; none | 3.1  [2.8-3.4] | 10.2  [9.6-10.8] | 18.9  [15.8-22.0] |  | 2.7  [2.5-3.0] | 9.1  [8.6-9.7] | 17.2  [14.0-20.3] | |  | | 0.2  [0.2-0.3] | 0.7  [0.6-0.9] | 1.2  [0.9-1.4] | |  | | 0.3  [0.2-0.3] | 0.8  [0.7-1.0] | 1.7  [1.4-2.0] | |
| 1-2; mild | 12.4  [11.5-13.4] | 51.5  [48.6-54.5] | 85.8  [78.6-93.0] |  | 10.3  [9.4-11.2] | 44.6  [41.9-47.4] | 71.5  [65.8-77.3] | |  | | 1.2  [0.9-1.5] | 4.0  [3.3-4.8] | 6.3  [5.2-7.4] | |  | | 2.1  [1.7-2.5] | 8.8  [7.6-10.0] | 17.3  [12.8-21.7] | |
| 3-4; moderate | 42.1  [37.7-46.4] | 192.4  [176.1-208.7] | 332.4  [298.8-365.8] |  | 35.0  [31.0-38.9] | 161.4  [146.3-176.4] | 281.8  [249.6-313.9] | |  | | 4.4  [3.0-5.8] | 15.0  [10.6-19.3] | 22.4  [16.2-28.5] | |  | | 10.4  [8.2-12.6] | 46.6  [38.6-54.6] | 80.7  [66.9-94.5] | |
| ≥5; severe | 76.3  [67.5-85.2] | 331.2  [299.7-362.7] | 605.5  [519.6-690.7] |  | 64.5  [56.3-72.7] | 284.7  [255.2-314.2] | 475.7  [416.9-534.2] | |  | | 5.9  [3.4-8.3] | 23.4  [15.1-31.7] | 58.9  [24.8-92.8] | |  | | 21.0  [16.3-25.7] | 90.9  [74.3-107.6] | 190.6  [132.8-248.0] | |
| Health conditions of interest | |  |  |  |  |  |  | |  | |  |  |  | |  | |  |  |  | |
| Dialysis |  |  |  |  |  |  |  | |  | |  |  |  | |  | |  |  |  | |
| Yes | 202.9  [132.1-273.1] | 685.7  [473.1-893.6] | 1070.2  [771.0-1359.7] |  | 142.8  [81.8-203.5] | 576.7  [373.7-775.3] | 965.6  [670.8-1251.0] | |  | | 42.0  [10.7-73.2] | 69.3  [20.2-118.2] | 145.7  [0.0-301.3] | |  | | 37.1  [11.0-63.2] | 150.0  [54.5-244.5] | 207.3  [83.3-329.8] | |
| No | 7.6  [7.2-7.9] | 25.2  [24.4-26.0] | 43.9  [40.5-47.2] |  | 6.4  [6.1-6.8] | 21.8  [21.1-22.6] | 37.0  [34.1-40.0] | |  | | 0.7  [0.6-0.8] | 1.9  [1.7-2.1] | 3.2  [2.5-3.9] | |  | | 1.3  [1.2-1.5] | 4.3  [3.9-4.6] | 8.4  [6.9-9.9] | |
| Pulmonary hypertension | |  |  |  |  |  |  | |  | |  |  |  | |  | |  |  |  | |
| Yes | 120.0  [68.0-171.7] | 522.1  [339.8-701.0] | 1087.7  [278.4-1829.7] |  | 90.1  [45.4-134.7] | 474.3  [297.0-648.4] | 988.6  [175.6-1734.4] | |  | | 19.0  [0.0-40.9] | 37.4  [0.0-79.5] | 65.5  [0.0-134.4] | |  | | 31.8  [6.3-57.1] | 71.4  [10.8-131.5] | 126.5  [29.0-223.1] | |
| No | 7.6  [7.3-8.0] | 25.3  [24.4-26.1] | 43.6  [40.3-46.8] |  | 6.5  [6.1-6.8] | 21.9  [21.1-22.7] | 36.7  [33.9-39.6] | |  | | 0.7  [0.6-0.8] | 1.9  [1.7-2.2] | 3.2  [2.5-4.0] | |  | | 1.3  [1.2-1.5] | 4.3  [4.0-4.7] | 8.4  [6.9-9.9] | |
| History of a transplant | |  |  |  |  |  |  | |  | |  |  |  | |  | |  |  |  | |
| Yes | 85.9  [65.7-105.9] | 240.1  [187.6-292.4] | 403.1  [202.8-599.2] |  | 65.3  [47.6-82.8] | 197.0  [148.5-245.2] | 264.0  [201.1-326.5] | |  | | 20.7  [10.9-30.5] | 46.0  [24.6-67.4] | 140.8  [0.0-325.2] | |  | | 14.1  [6.4-21.8] | 37.4  [17.1-57.7] | 69.4  [33.4-105.2] | |
| No | 7.4  [7.1-7.8] | 25.0  [24.1-25.8] | 43.3  [40.0-46.6] |  | 6.3  [6.0-6.6] | 21.6  [20.9-22.4] | 36.9  [33.9-39.8] | |  | | 0.6  [0.5-0.7] | 1.8  [1.6-2.0] | 2.8  [2.5-3.2] | |  | | 1.3  [1.2-1.5] | 4.3  [3.9-4.6] | 8.3  [6.8-9.8] | |
| Dementia |  |  |  |  |  |  |  | |  | |  |  |  | |  | |  |  |  | |
| Yes | 68.9  [59.8-78.0] | 356.7  [311.9-401.3] | 804.0  [693.7-912.9] |  | 53.7  [45.6-61.7] | 265.8  [226.6-304.8] | 560.2  [477.1-642.6] | |  | | 2.0  [0.4-3.6] | 15.2  [4.6-25.7] | 21.2  [7.8-34.6] | |  | | 23.3  [18.0-28.5] | 124.8  [98.8-150.7] | 340.5  [259.0-421.4] | |
| No | 6.9  [6.5-7.2] | 23.1  [22.3-23.9] | 39.0  [36.0-42.0] |  | 5.9  [5.6-6.2] | 20.3  [19.6-21.1] | 34.2  [31.4-37.1] | |  | | 0.7  [0.6-0.8] | 1.9  [1.7-2.1] | 3.2  [2.5-3.9] | |  | | 1.1  [0.9-1.2] | 3.4  [3.1-3.7] | 5.8  [5.2-6.4] | |
| Pregnancy |  |  |  |  |  |  |  | |  | |  |  |  | |  | |  |  |  | |
| Yes | 62.5  [42.1-82.8] | 78.2  [54.2-102.1] | 85.1  [59.3-110.8] |  | 62.5  [42.1-82.8] | 78.2  [54.2-102.1] | 85.1  [59.3-110.8] | |  | | 0.0  [0.0-0.0] | 0.0  [0.0-0.0] | 0.0  [0.0-0.0] | |  | | 0.0  [0.0-0.0] | 0.0  [0.0-0.0] | 0.0  [0.0-0.0] | |
| No | 7.6  [7.2-7.9] | 25.4  [24.6-26.2] | 44.2  [40.9-47.6] |  | 6.4  [6.1-6.7] | 22.0  [21.2-22.7] | 37.3  [34.3-40.3] | |  | | 0.7  [0.6-0.8] | 2.0  [1.7-2.2] | 3.3  [2.6-4.0] | |  | | 1.4  [1.2-1.5] | 4.4  [4.0-4.7] | 8.5  [7.0-10.0] | |
| Cancer |  |  |  |  |  |  |  | |  | |  |  |  | |  | |  |  |  | |
| Yes | 53.2  [45.3-61.0] | 181.9  [157.0-206.7] | 282.4  [246.5-318.3] |  | 43.3  [36.3-50.4] | 151.7  [128.9-174.5] | 233.7  [200.8-266.4] | |  | | 7.5  [4.5-10.4] | 14.5  [8.0-21.1] | 26.9  [15.5-38.2] | |  | | 15.4  [11.1-19.8] | 51.0  [37.7-64.3] | 83.3  [63.9-102.8] | |
| No | 7.1  [6.7-7.4] | 24.0  [23.2-24.8] | 42.3  [38.9-45.7] |  | 6.0  [5.7-6.3] | 20.9  [20.1-21.6] | 35.8  [32.8-38.8] | |  | | 0.6  [0.5-0.7] | 1.8  [1.6-2.0] | 3.1  [2.4-3.8] | |  | | 1.2  [1.0-1.3] | 3.9  [3.6-4.2] | 7.9  [6.4-9.3] | |
| Chronic obstructive pulmonary disease | | |  |  |  |  |  | |  | |  |  |  | |  | |  |  |  | |
| Yes | 40.4  [36.6-44.2] | 168.6  [156.4-180.9] | 306.7  [273.7-339.6] |  | 34.4  [30.9-37.9] | 145.8  [134.3-157.3] | 255.5  [228.6-282.4] | |  | | 3.2  [2.1-4.2] | 12.0  [8.8-15.2] | 20.7  [15.7-25.6] | |  | | 10.3  [8.4-12.3] | 42.5  [36.3-48.7] | 81.1  [60.9-101.4] | |
| No | 6.2  [5.8-6.5] | 20.4  [19.7-21.2] | 35.2  [32.1-38.2] |  | 5.2  [4.9-5.5] | 17.7  [17.0-18.4] | 29.9  [27.1-32.7] | |  | | 0.6  [0.5-0.7] | 1.6  [1.4-1.8] | 2.7  [2.0-3.5] | |  | | 0.9  [0.8-1.1] | 3.0  [2.7-3.3] | 5.9  [4.7-7.0] | |
| Chronic kidney disease | |  |  |  |  |  |  | |  | |  |  |  | |  | |  |  |  | |
| Yes | 39.5  [36.0-43.0] | 178.4  [165.6-191.1] | 319.7  [289.7-349.6] |  | 32.5  [29.3-35.7] | 151.1  [139.3-162.9] | 262.0  [238.7-285.2] | |  | | 3.8  [2.7-4.8] | 14.2  [10.7-17.7] | 28.1  [17.4-38.9] | |  | | 9.4  [7.7-11.2] | 43.9  [37.5-50.3] | 86.8  [68.3-105.1] | |
| No | 6.0  [5.6-6.3] | 19.9  [19.2-20.7] | 34.4  [31.3-37.5] |  | 5.1  [4.8-5.4] | 17.4  [16.7-18.1] | 29.7  [26.8-32.6] | |  | | 0.5  [0.4-0.6] | 1.5  [1.3-1.7] | 2.3  [2.0-2.6] | |  | | 0.9  [0.8-1.0] | 2.9  [2.6-3.2] | 5.5  [4.4-6.6] | |
| Parkinson's disease | |  |  |  |  |  |  | |  | |  |  |  | |  | |  |  |  | |
| Yes | 38.6  [23.4-53.7] | 284.5  [195.3-372.9] | 918.5  [488.5-1329.1] |  | 27.9  [15.0-40.8] | 258.8  [172.2-344.7] | 785.3  [375.2-1177.9] | |  | | 1.7  [0.0-4.9] | 11.7  [0.0-31.6] | 27.2  [0.0-63.4] | |  | | 14.2  [4.9-23.6] | 49.8  [17.0-82.5] | 216.2  [53.7-376.1] | |
| No | 7.6  [7.3-8.0] | 25.2  [24.3-26.0] | 42.5  [39.4-45.6] |  | 6.5  [6.1-6.8] | 21.8  [21.0-22.6] | 35.9  [33.2-38.5] | |  | | 0.7  [0.6-0.8] | 1.9  [1.7-2.2] | 3.3  [2.5-4.0] | |  | | 1.3  [1.2-1.5] | 4.3  [3.9-4.6] | 8.1  [6.7-9.5] | |
| Cardiovascular disease | |  |  |  |  |  |  | |  | |  |  |  | |  | |  |  |  | |
| Yes | 31.1  [28.9-33.4] | 119.5  [113.1-126.0] | 203.5  [185.4-221.5] |  | 25.8  [23.8-27.9] | 101.7  [95.7-107.7] | 165.4  [151.2-179.6] | |  | | 2.9  [2.3-3.6] | 9.5  [7.7-11.2] | 17.0  [11.6-22.3] | |  | | 7.6  [6.5-8.7] | 26.7  [23.7-29.7] | 49.9  [39.4-60.4] | |
| No | 5.1  [4.8-5.4] | 16.9  [16.2-17.6] | 29.5  [26.6-32.4] |  | 4.4  [4.1-4.7] | 14.8  [14.2-15.5] | 25.9  [23.1-28.7] | |  | | 0.4  [0.4-0.5] | 1.2  [1.1-1.4] | 1.9  [1.7-2.2] | |  | | 0.7  [0.5-0.8] | 2.3  [2.0-2.5] | 4.4  [3.7-5.2] | |
| Other immunocompromising conditions | | |  |  |  |  |  | |  | |  |  |  | |  | |  |  |  | |
| Yes | 26.1  [23.8-28.5] | 85.6  [79.7-91.6] | 128.5  [113.9-143.0] |  | 20.8  [18.7-22.9] | 72.0  [66.5-77.5] | 105.7  [93.5-117.9] | |  | | 3.6  [2.8-4.5] | 8.5  [6.7-10.2] | 15.9  [8.3-23.5] | |  | | 6.4  [5.3-7.6] | 17.5  [14.8-20.1] | 24.8  [21.3-28.2] | |
| No | 6.2  [5.8-6.5] | 21.1  [20.3-21.9] | 38.3  [34.9-41.7] |  | 5.3  [5.0-5.6] | 18.5  [17.7-19.2] | 32.6  [29.5-35.6] | |  | | 0.5  [0.4-0.5] | 1.5  [1.3-1.7] | 2.3  [2.0-2.6] | |  | | 0.9  [0.8-1.1] | 3.4  [3.1-3.7] | 7.3  [5.7-8.9] | |
| Diabetes |  |  |  |  |  |  |  | |  | |  |  |  | |  | |  |  |  | |
| Yes | 24.8  [22.8-26.8] | 105.1  [98.6-111.5] | 172.5  [156.1-188.9] |  | 20.6  [18.8-22.4] | 89.6  [83.6-95.6] | 140.5  [128.4-152.6] | |  | | 2.8  [2.1-3.4] | 9.3  [7.5-11.2] | 17.7  [11.6-23.8] | |  | | 4.6  [3.7-5.5] | 20.6  [17.7-23.4] | 38.4  [28.3-48.5] | |
| No | 5.8  [5.4-6.1] | 18.8  [18.1-19.6] | 33.5  [30.2-36.7] |  | 4.9  [4.6-5.2] | 16.4  [15.7-17.1] | 28.9  [25.9-32.0] | |  | | 0.5  [0.4-0.6] | 1.3  [1.1-1.5] | 2.0  [1.7-2.3] | |  | | 1.0  [0.9-1.1] | 3.0  [2.7-3.3] | 5.8  [4.7-7.0] | |
| Immunocompromised (overall) | | |  |  |  |  |  | |  | |  |  |  | |  | |  |  |  | |
| Yes | 24.0  [22.0-25.9] | 79.9  [74.8-85.1] | 120.8  [108.6-133.1] |  | 19.2  [17.4-21.0] | 67.6  [62.8-72.4] | 100.5  [90.2-110.8] | |  | | 3.1  [2.4-3.8] | 7.4  [5.9-8.9] | 14.1  [7.7-20.5] | |  | | 5.7  [4.8-6.7] | 16.0  [13.7-18.2] | 23.7  [20.5-26.8] | |
| No | 6.0  [5.6-6.3] | 20.5  [19.7-21.2] | 37.4  [33.9-40.9] |  | 5.2  [4.9-5.5] | 17.9  [17.1-18.6] | 31.8  [28.7-34.9] | |  | | 0.4  [0.4-0.5] | 1.4  [1.2-1.6] | 2.2  [1.9-2.5] | |  | | 0.9  [0.8-1.0] | 3.3  [2.9-3.6] | 7.1  [5.5-8.7] | |
| Hypertension | |  |  |  |  |  |  | |  | |  |  |  | |  | |  |  |  | |
| Yes | 18.4  [17.2-19.5] | 82.9  [78.9-86.9] | 142.5  [132.2-152.9] |  | 15.2  [14.2-16.3] | 71.3  [67.6-75.0] | 120.3  [111.3-129.3] | |  | | 1.8  [1.5-2.2] | 6.2  [5.2-7.2] | 10.9  [8.0-13.8] | |  | | 4.1  [3.6-4.7] | 18.2  [16.4-20.1] | 31.5  [26.7-36.3] | |
| No | 4.6  [4.3-4.9] | 14.4  [13.7-15.0] | 25.2  [22.1-28.2] |  | 4.0  [3.7-4.3] | 12.6  [12.0-13.3] | 21.5  [18.8-24.2] | |  | | 0.4  [0.3-0.5] | 1.1  [0.9-1.3] | 1.7  [1.4-1.9] | |  | | 0.6  [0.4-0.7] | 1.6  [1.4-1.9] | 4.1  [2.7-5.4] | |
| Autoimmune disorders | |  |  |  |  |  |  | |  | |  |  |  | |  | |  |  |  | |
| Yes | 17.2  [14.2-20.1] | 60.7  [52.8-68.7] | 105.3  [73.8-136.7] |  | 12.8  [10.3-15.3] | 51.0  [43.6-58.3] | 92.9  [61.6-124.0] | |  | | 2.8  [1.6-4.0] | 6.5  [4.0-8.9] | 8.4  [5.3-11.4] | |  | | 4.1  [2.6-5.5] | 9.6  [6.6-12.7] | 15.0  [10.5-19.5] | |
| No | 7.4  [7.0-7.7] | 24.5  [23.7-25.4] | 42.6  [39.3-46.0] |  | 6.3  [6.0-6.6] | 21.3  [20.5-22.1] | 35.9  [33.0-38.8] | |  | | 0.6  [0.5-0.7] | 1.8  [1.6-2.0] | 3.1  [2.4-3.9] | |  | | 1.3  [1.1-1.4] | 4.2  [3.9-4.5] | 8.3  [6.8-9.8] | |
| Obesity |  |  |  |  |  |  |  | |  | |  |  |  | |  | |  |  |  | |
| Yes | 15.8  [14.0-17.5] | 53.9  [49.6-58.2] | 89.9  [74.9-104.8] |  | 12.8  [11.2-14.3] | 46.0  [42.0-50.1] | 74.9  [61.8-88.0] | |  | | 2.0  [1.4-2.7] | 5.6  [4.3-7.0] | 12.0  [4.9-19.1] | |  | | 2.9  [2.2-3.7] | 8.5  [6.8-10.2] | 12.3  [9.8-14.8] | |
| No | 7.0  [6.6-7.3] | 23.1  [22.2-23.9] | 40.4  [37.0-43.7] |  | 5.9  [5.6-6.3] | 20.0  [19.3-20.8] | 34.2  [31.2-37.2] | |  | | 0.6  [0.5-0.7] | 1.6  [1.4-1.8] | 2.5  [2.2-2.8] | |  | | 1.2  [1.1-1.4] | 4.0  [3.6-4.3] | 8.1  [6.5-9.8] | |
| Mental health disorder | | |  |  |  |  |  | |  | |  |  |  | |  | |  |  |  | |
| Yes | 13.9  [12.7-15.1] | 46.5  [43.7-49.4] | 87.1  [71.0-103.1] |  | 11.9  [10.8-13.0] | 40.4  [37.7-43.1] | 76.6  [60.9-92.3] | |  | | 1.2  [0.9-1.6] | 3.7  [2.9-4.5] | 5.3  [4.3-6.4] | |  | | 2.2  [1.7-2.7] | 6.3  [5.3-7.4] | 11.1  [8.0-14.3] | |
| No | 6.6  [6.2-6.9] | 21.7  [20.8-22.5] | 36.8  [33.9-39.6] |  | 5.6  [5.2-5.9] | 18.7  [17.9-19.5] | 30.5  [28.2-32.8] | |  | | 0.6  [0.5-0.7] | 1.6  [1.4-1.8] | 2.9  [2.1-3.7] | |  | | 1.2  [1.1-1.4] | 4.0  [3.6-4.3] | 8.0  [6.3-9.6] | |
| Down's syndrome | |  |  |  |  |  |  | |  | |  |  |  | |  | |  |  |  | |
| Yes | 12.4  [0.0-36.7] | 201.4  [54.5-346.0] | 282.7  [97.9-464.0] |  | 12.4  [0.0-36.7] | 147.6  [23.6-269.9] | 229.3  [61.5-394.3] | |  | | 0.0  [0.0-0.0] | 17.4  [0.0-51.3] | 17.4  [0.0-51.3] | |  | | 0.0  [0.0-0.0] | 68.9  [0.0-163.8] | 68.9  [0.0-163.8] | |
| No | 7.7  [7.4-8.1] | 25.5  [24.6-26.3] | 44.2  [40.9-47.6] |  | 6.5  [6.2-6.9] | 22.1  [21.3-22.8] | 37.4  [34.4-40.3] | |  | | 0.7  [0.6-0.8] | 1.9  [1.7-2.2] | 3.3  [2.6-4.0] | |  | | 1.4  [1.2-1.5] | 4.3  [4.0-4.7] | 8.5  [7.0-9.9] | |
| Asthma |  |  |  |  |  |  |  | |  | |  |  |  | |  | |  |  |  | |
| Yes | 12.2  [9.9-14.5] | 48.3  [42.0-54.6] | 67.3  [59.0-75.5] |  | 10.7  [8.5-12.8] | 43.5  [37.5-49.5] | 61.2  [53.2-69.1] | |  | | 1.1  [0.4-1.8] | 3.7  [2.0-5.4] | 5.0  [2.8-7.1] | |  | | 1.3  [0.6-2.1] | 5.8  [3.6-8.1] | 7.1  [4.5-9.6] | |
| No | 7.5  [7.2-7.9] | 24.7  [23.9-25.5] | 43.5  [40.0-46.9] |  | 6.4  [6.0-6.7] | 21.3  [20.5-22.1] | 36.6  [33.5-39.6] | |  | | 0.7  [0.6-0.8] | 1.9  [1.7-2.1] | 3.2  [2.5-3.9] | |  | | 1.4  [1.2-1.5] | 4.3  [4.0-4.6] | 8.5  [7.0-10.0] | |
| Dyslipidemia | |  |  |  |  |  |  | |  | |  |  |  | |  | |  |  |  | |
| Yes | 8.2  [7.5-9.0] | 33.0  [30.8-35.2] | 55.4  [48.7-62.1] |  | 6.8  [6.1-7.4] | 28.7  [26.6-30.7] | 46.5  [41.4-51.6] | |  | | 1.0  [0.8-1.3] | 3.0  [2.3-3.6] | 4.4  [3.6-5.3] | |  | | 1.6  [1.2-1.9] | 5.4  [4.5-6.3] | 11.1  [6.6-15.5] | |
| No | 7.6  [7.2-8.0] | 23.8  [22.9-24.6] | 41.8  [37.9-45.6] |  | 6.5  [6.1-6.8] | 20.6  [19.7-21.4] | 35.4  [31.9-38.9] | |  | | 0.6  [0.5-0.7] | 1.7  [1.5-1.9] | 3.0  [2.1-3.9] | |  | | 1.3  [1.1-1.5] | 4.1  [3.7-4.5] | 7.8  [6.4-9.2] | |
|  |  |  |  |  |  |  |  | |  | |  |  |  | |  | |  |  |  | |
| **Other factors** |  |  |  |  |  |  |  | |  | |  |  |  | |  | |  |  |  | |
| Prior SARS-CoV-2 infection | |  |  |  |  |  |  | |  | |  |  |  | |  | |  |  |  | |
| Yes | 7.3  [5.8-8.7] | 13.6  [11.4-15.8] | 29.7  [10.8-48.6] |  | 5.8  [4.5-7.1] | 11.1  [9.1-13.0] | 16.4  [13.6-19.1] | |  | | 0.4  [0.0-0.7] | 1.2  [0.5-1.8] | 2.0  [0.9-3.1] | |  | | 1.3  [0.7-1.9] | 2.0  [1.1-2.8] | 12.4  [0.0-31.1] | |
| No | 7.7  [7.4-8.1] | 26.5  [25.6-27.4] | 45.5  [42.2-48.9] |  | 6.6  [6.2-6.9] | 23.0  [22.2-23.8] | 39.0  [35.8-42.1] | |  | | 0.7  [0.6-0.8] | 2.0  [1.8-2.2] | 3.4  [2.6-4.1] | |  | | 1.4  [1.2-1.5] | 4.5  [4.2-4.9] | 8.4  [7.1-9.6] | |
| Received nirmatrelvir/ritonavir | | |  |  |  |  |  | |  | |  |  |  | |  | |  |  |  | |
| Yes | 18.1  [11.5-24.7] | 149.2  [108.9-189.4] | 305.1  [230.7-378.8] |  | 15.2  [9.2-21.3] | 138.7  [99.1-178.1] | 274.1  [203.0-344.7] | |  | | 2.9  [0.3-5.4] | 6.6  [0.9-12.3] | 19.5  [0.0-39.0] | |  | | 0.6  [0.0-1.8] | 8.4  [0.0-17.9] | 36.0  [9.4-62.5] | |
| No | 7.6  [7.3-8.0] | 25.1  [24.3-25.9] | 43.6  [40.2-46.9] |  | 6.5  [6.1-6.8] | 21.7  [20.9-22.5] | 36.7  [33.8-39.7] | |  | | 0.7  [0.6-0.8] | 1.9  [1.7-2.1] | 3.2  [2.5-3.9] | |  | | 1.4  [1.2-1.5] | 4.3  [4.0-4.7] | 8.4  [6.9-9.9] | |
| Early vaccination | |  |  |  |  |  |  | |  | |  |  |  | |  | |  |  |  | |
| Yes | 7.1  [6.8-7.5] | 26.4  [25.5-27.3] | 45.7  [42.3-49.0] |  | 6.0  [5.7-6.4] | 22.9  [22.1-23.8] | 38.6  [35.6-41.6] | |  | | 0.6  [0.5-0.7] | 2.0  [1.7-2.2] | 3.3  [2.6-4.0] | |  | | 1.3  [1.1-1.4] | 4.6  [4.2-5.0] | 8.8  [7.3-10.3] | |
| No | 15.5  [13.7-17.4] | 24.1  [21.7-26.4] | 45.7  [42.3-49.0] |  | 13.1  [11.4-14.8] | 20.4  [18.3-22.6] | 38.6  [35.6-41.6] | |  | | 1.7  [1.1-2.3] | 2.4  [1.7-3.1] | 3.3  [2.6-4.0] | |  | | 2.2  [1.5-2.9] | 3.3  [2.5-4.2] | 8.8  [7.3-10.3] | |
| Pandemic wave vaccine dose received* | | |  |  |  |  |  | |  | |  |  |  | |  | |  |  |  | |
| Wave 2 | 9.5  [4.4-14.7] | 45.1  [21.2-68.9] | 138.5  [80.3-196.3] |  | 3.7  [0.5-6.9] | 27.5  [6.3-48.6] | 66.1  [26.0-106.1] | |  | | 0.0  [0.0-0.0] | 4.6  [0.0-13.7] | 4.6  [0.0-13.7] | |  | | 7.3  [2.8-11.9] | 21.2  [8.1-34.3] | 98.5  [48.6-148.1] | |
| Wave 3 (Alpha) | 6.2  [5.8-6.6] | 38.3  [36.4-40.2] | 64.8  [60.7-68.8] |  | 5.1  [4.7-5.5] | 32.7  [30.9-34.4] | 54.9  [51.2-58.7] | |  | | 0.6  [0.5-0.8] | 3.1  [2.6-3.6] | 4.8  [3.9-5.8] | |  | | 1.5  [1.3-1.7] | 8.3  [7.4-9.2] | 13.7  [12.1-15.3] | |
| Wave 4 (Delta) | 8.9  [8.3-9.5] | 20.2  [19.2-21.2] | 138.5  [80.3-196.3] |  | 7.8  [7.3-8.4] | 18.2  [17.2-19.1] | 66.1  [26.0-106.1] | |  | | 0.7  [0.5-0.8] | 1.3  [1.1-1.6] | 4.6  [0.0-13.7] | |  | | 1.0  [0.8-1.2] | 2.1  [1.8-2.4] | 98.5  [48.6-148.1] | |
| Wave 5 (Omicron) | 16.1  [13.7-18.4] | 26.2  [23.2-29.2] | 64.8  [60.7-68.8] |  | 13.1  [11.0-15.2] | 22.2  [19.4-25.0] | 54.9  [51.2-58.7] | |  | | 1.7  [0.9-2.4] | 2.4  [1.5-3.4] | 4.8  [3.9-5.8] | |  | | 2.8  [1.8-3.7] | 3.9  [2.7-5.1] | 13.7  [12.1-15.3] | |

*Pandemic wave was measured on the day follow-up commenced. Abbreviations: CI = confidence interval; COVID-19 = coronavirus disease 2019; SARS-CoV-2 = severe acute respiratory syndrome coronavirus 2.
