# Supplementary material for: Factors associated with severe COVID-19 outcomes among adults with at least a primary vaccination schedule: A retrospective cohort study from Alberta, Canada
Source: PLoS One. 2026 Aug 17;21(8):e0354822. doi: 10.1371/journal.pone.0354822 (PMC13480603; doi:10.1371/journal.pone.0354822)
Supplement: Supplementary file 7 [file pone.0354822.s007.docx]

# Supplementary Table 6. Unadjusted likelihood of a severe COVID-19 outcome among adults who completed a primary vaccination series, presented according to the type of severe COVID-19 outcome.

|  | Type of severe COVID-19 outcome | | |
| --- | --- | --- | --- |
|  | Hospitalization  (non-intensive care unit) | Intensive care unit admission | Death |
|  | unadjusted  hazard ratio [95% CI] | unadjusted  hazard ratio [95% CI] | unadjusted  hazard ratio [95% CI] |
| **Demographic characteristics** |  |  |  |
| Age (years) |  |  |  |
| Continuous (reference: one year lower) | 1.07 [1.06-1.07] | 1.05 [1.05-1.06] | 1.12 [1.12-1.13] |
| Category |  |  |  |
| 18-39 (reference) | - | - | - |
| 40-49 | 0.94 [0.83-1.07] | 2.68 [1.81-3.96] | 2.55 [1.44-4.53] |
| 50-59 | 1.88 [1.68-2.10] | 4.55 [3.15-6.57] | 8.84 [5.45-14.32] |
| 60-64 | 3.33 [2.94-3.77] | 9.70 [6.63-14.18] | 21.35 [13.11-34.77] |
| ≥65 | 11.37 [10.47-12.35] | 12.78 [9.18-17.80] | 138.49 [90.48-211.99] |
| Older age |  |  |  |
| ≥65 (reference: <65) | 8.61 [8.09-9.16] | 4.65 [3.79-5.69] | 33.21 [28.05-39.31] |
| Sex |  |  |  |
| Male (reference: female) | 1.03 [0.97-1.09] | 1.69 [1.39-2.07] | 1.43 [1.25-1.63] |
| Residence |  |  |  |
| Rural (reference: urban) | 1.49 [1.38-1.62] | 2.33 [1.86-2.92] | 1.66 [1.40-1.96] |
| Long-term care (reference: no) | 3.62 [2.65-4.94] | 0.85 [0.12-6.03] | 44.72 [35.56-56.24] |
|  |  |  |  |
| **Socioeconomic status** |  |  |  |
| Material deprivation index |  |  |  |
| 1 (most well off; reference) | - | - | - |
| 2 | 0.97 [0.87-1.09] | 0.73 [0.50-1.06] | 1.13 [0.86-1.49] |
| 3 | 1.12 [1.00-1.24] | 1.10 [0.79-1.52] | 1.54 [1.20-1.97] |
| 4 | 1.37 [1.24-1.51] | 1.23 [0.90-1.68] | 2.02 [1.60-2.54] |
| 5 (most deprived) | 1.75 [1.59-1.92] | 1.39 [1.03-1.88] | 2.05 [1.63-2.58] |
|  |  |  |  |
| **Clinical characteristics** |  |  |  |
| Charlson Comorbidity Index |  |  |  |
| Continuous (reference: one score lower) | 1.51 [1.50-1.52] | 1.49 [1.45-1.54] | 1.60 [1.58-1.63] |
| Category |  |  |  |
| 0; no comorbid burden (reference) | - | - | - |
| 1-2; mild comorbid burden | 4.69 [4.36-5.04] | 5.47 [4.30-6.95] | 9.02 [7.43-10.95] |
| 3-4; moderate comorbid burden | 17.05 [15.59-18.63] | 19.84 [14.89-26.45] | 47.25 [38.38-58.18] |
| ≥5; severe comorbid burden | 30.82 [27.98-33.94] | 32.18 [23.36-44.33] | 98.42 [79.60-121.71] |
| Health conditions of interest |  |  |  |
| (reference: not living with the condition) |  |  |  |
| Dialysis | 27.09 [20.80-35.27] | 51.56 [28.28-94.00] | 36.94 [22.52-60.62] |
| Pulmonary hypertension | 17.70 [13.01-24.07] | 21.39 [8.85-51.70] | 20.53 [11.00-38.29] |
| Dementia | 11.92 [10.75-13.22] | 3.96 [2.36-6.64] | 35.35 [30.10-41.53] |
| History of a transplant | 9.51 [7.80-11.60] | 25.71 [17.26-38.28] | 10.98 [7.32-16.48] |
| Parkinson's disease | 9.24 [7.28-11.73] | 3.89 [1.25-12.11] | 14.29 [9.35-21.84] |
| Chronic kidney disease | 8.38 [7.82-8.99] | 9.04 [7.25-11.27] | 14.06 [12.23-16.15] |
| Chronic obstructive pulmonary disease | 8.22 [7.65-8.83] | 7.33 [5.79-9.28] | 13.31 [11.56-15.32] |
| Cancer | 7.56 [6.71-8.51] | 9.94 [7.10-13.90] | 13.40 [10.90-16.47] |
| Cardiovascular disease | 6.74 [6.34-7.17] | 7.65 [6.28-9.32] | 11.47 [10.06-13.08] |
| Down's syndrome | 5.54 [2.77-11.08] | 7.03 [0.99-50.01] | 6.65 [1.66-26.65] |
| Hypertension | 5.32 [5.00-5.65] | 5.47 [4.49-6.65] | 9.54 [8.30-10.97] |
| Diabetes | 5.00 [4.68-5.33] | 7.15 [5.86-8.73] | 6.00 [5.22-6.89] |
| Other immunocompromising conditions | 3.81 [3.55-4.10] | 6.13 [4.97-7.56] | 5.17 [4.47-6.00] |
| Immunocompromised (overall) | 3.73 [3.48-3.99] | 5.49 [4.48-6.74] | 4.98 [4.32-5.73] |
| Pregnancy | 3.80 [2.82-5.12] | NA* | NA* |
| Autoimmune disorders | 2.39 [2.12-2.71] | 3.51 [2.52-4.91] | 2.47 [1.90-3.22] |
| Obesity | 2.25 [2.07-2.44] | 3.55 [2.82-4.45] | 1.99 [1.66-2.40] |
| Mental health disorder | 2.15 [2.01-2.30] | 2.16 [1.75-2.68] | 1.60 [1.36-1.87] |
| Asthma | 1.91 [1.69-2.16] | 1.77 [1.18-2.65] | 1.08 [0.77-1.53] |
| Dyslipidemia | 1.32 [1.23-1.41] | 1.76 [1.43-2.16] | 1.29 [1.11-1.51] |
|  |  |  |  |
| **Other factors** |  |  |  |
| Prior SARS-CoV-2 infection  (reference: no) | 0.53 [0.45-0.62] | 0.58 [0.35-0.96] | 0.48 [0.34-0.70] |

*The number of events were not sufficient to calculate the hazard ratio. Abbreviations: CI = confidence interval; COVID-19 = coronavirus disease 2019; NA = not applicable; SARS-CoV-2 = severe acute respiratory syndrome coronavirus 2.
