# Supplementary material for: Factors associated with severe COVID-19 outcomes among adults with at least a primary vaccination schedule: A retrospective cohort study from Alberta, Canada
Source: PLoS One. 2026 Aug 17;21(8):e0354822. doi: 10.1371/journal.pone.0354822 (PMC13480603; doi:10.1371/journal.pone.0354822)
Supplement: Supplementary file 8 [file pone.0354822.s008.docx]

# Supplementary Table 7. Unadjusted risk profiles among adults who completed a primary vaccination series.

|  | People who had a severe  COVID-19 outcome | Risk profile  population size | Hazard Ratio  Estimate | Population attributable risk |
| --- | --- | --- | --- | --- |
|  | n (%) | n (% of cohort) | unadjusted  hazard ratio [95% CI] | % |
| **Hospitalization (non-intensive care unit admission)** | *n=4,703* |  |  |  |
| Age ≥75 | 1,619 (34.4%) | 219,855 (8.3%) | 11.34 [10.65-12.08] | 28.5% |
| Age ≥65 | 2,509 (54.0%) | 553,854 (21.0%) | 8.60 [8.10-9.12] | 41.8% |
| Age ≥65, or hypertension | 3,163 (67.3%) | 846,335 (32.1%) | 7.10 [6.67-7.55] | 51.8% |
| Age ≥65, or CVD | 3,098 (65.9%) | 695,928 (26.4%) | 8.88 [8.35-9.44] | 53.7% |
| Age ≥65, or CVD, or COPD | 3,030 (64.4%) | 648,368 (24.6%) | 9.93 [9.34-10.56] | 52.8% |
| Age ≥65, or CVD, or diabetes | 3,367 (71.6%) | 815,744 (30.9%) | 8.82 [8.27-9.40] | 58.9% |
| Age ≥65, or CVD, or obesity | 3,319 (70.6%) | 840,815 (31.9%) | 7.67 [7.20-8.17] | 56.8% |
| Age ≥65, or CVD, or diabetes, or obesity | 3,514 (74.7%) | 933,539 (35.4%) | 7.92 [7.41-8.46] | 60.9% |
| Age ≥65, or any health condition of interest* | 4,703 (89.1%) | 1,442,900 (54.7%) | 8.90 [8.12-9.76] | 75.9% |
| CVD, or COPD | 2,285 (48.6%) | 361,096 (13.7%) | 7.58 [7.15-8.03] | 40.4% |
| Any health condition of interest* | 4,703 (86.2%) | 1,330,809 (50.4%) | 7.63 [7.02-8.29] | 72.2% |
| Immunocompromised status, or CKD | 2,044 (43.5%) | 414,626 (15.7%) | 5.30 [5.00-5.62] | 32.9% |
| Immunocompromised status, or CKD, or LTC | 2,067 (44.0%) | 422,279 (16.0%) | 5.33 [5.03-5.65] | 33.3% |
|  |  |  |  |  |
| **Intensive care unit admission** | *n=432* |  |  |  |
| Age ≥75 | 61 (14.1%) | 219,855 (8.3%) | 3.00 [2.28-3.96] | 6.3% |
| Age ≥65 | 177 (41.0%) | 553,854 (21.0%) | 4.54 [3.72-5.54] | 25.3% |
| Age ≥65, or hypertension | 282 (65.3%) | 846,335 (32.1%) | 6.16 [5.04-7.53] | 48.9% |
| Age ≥65, or CVD | 267 (61.8%) | 695,928 (26.4%) | 7.02 [5.76-8.55] | 48.1% |
| Age ≥65, or CVD, or COPD | 250 (57.9%) | 648,368 (24.6%) | 6.99 [5.75-8.50] | 44.2% |
| Age ≥65, or CVD, or diabetes | 316 (73.1%) | 815,744 (30.9%) | 9.11 [7.35-11.30] | 61.1% |
| Age ≥65, or CVD, or obesity | 293 (67.8%) | 840,815 (31.9%) | 6.45 [5.26-7.91] | 52.8% |
| Age ≥65, or CVD, or diabetes, or obesity | 331 (76.6%) | 933,539 (35.4%) | 8.45 [6.75-10.58] | 63.8% |
| Age ≥65, or any health condition of interest* | 432 (90.0%) | 1,442,900 (54.7%) | 9.62 [7.02-13.19] | 78.0% |
| CVD, or COPD | 211 (48.8%) | 361,096 (13.7%) | 7.45 [6.16-9.00] | 40.7% |
| Any health condition of interest* | 432 (88.2%) | 1,330,809 (50.4%) | 8.92 [6.65-11.95] | 76.2% |
| Immunocompromised status, or CKD | 206 (47.7%) | 414,626 (15.7%) | 6.13 [5.07-7.41] | 37.9% |
| Immunocompromised status, or CKD, or LTC | 207 (47.9%) | 422,279 (16.0%) | 6.09 [5.04-7.36] | 38.0% |
|  |  |  |  |  |
| **Death** | *n=970* |  |  |  |
| Age ≥75 | 570 (58.8%) | 846,335 (32.1%) | 33.72 [29.49-38.56] | 55.0% |
| Age ≥65 | 782 (80.6%) | 553,854 (21.0%) | 32.49 [27.62-38.22] | 75.5% |
| Age ≥65, or hypertension | 859 (88.6%) | 422,279 (16.0%) | 27.32 [22.39-33.33] | 83.2% |
| Age ≥65, or CVD | 855 (88.1%) | 695,928 (26.4%) | 35.03 [28.80-42.61] | 83.9% |
| Age ≥65, or CVD, or COPD | 841 (86.7%) | 361,096 (13.7%) | 36.96 [30.65-44.56] | 82.4% |
| Age ≥65, or CVD, or diabetes | 877 (90.4%) | 695,928 (26.4%) | 33.49 [27.02-41.51] | 86.1% |
| Age ≥65, or CVD, or obesity | 877 (90.4%) | 815,744 (30.9%) | 30.55 [24.65-37.86] | 85.9% |
| Age ≥65, or CVD, or diabetes, or obesity | 889 (91.6%) | 840,815 (31.9%) | 29.66 [23.61-37.26] | 87.1% |
| Age ≥65, or any health condition of interest* | 970 (96.4%) | 1,442,900 (54.7%) | 29.18 [20.82-40.90] | 92.0% |
| CVD, or COPD | 613 (63.2%) | 1,417,763 (53.7%) | 13.77 [12.08-15.70] | 57.4% |
| Any health condition of interest* | 970 (92.8%) | 1,330,809 (50.4%) | 15.66 [12.28-19.98] | 85.4% |
| Immunocompromised status, or CKD | 542 (55.9%) | 648,368 (24.6%) | 8.74 [7.69-9.92] | 47.7% |
| Immunocompromised status, or CKD, or LTC | 585 (60.3%) | 414,626 (15.7%) | 10.35 [9.10-11.78] | 52.8% |

*Health conditions of interest included asthma, cardiovascular disease, chronic kidney disease, chronic obstructive pulmonary disease, dementia, diabetes, Down's syndrome, hypertension, an immunocompromised status, a mental health disorder, obesity, Parkinson's disease, pregnancy, and pulmonary hypertension; dyslipidemia was not included. Hazard ratio estimates are unadjusted, with the reference group comprising individuals without the specified condition(s). Population attributable risk is unadjusted and based on observed incidence and risk factor prevalence in the cohort, estimating the proportion of severe COVID-19 outcomes potentially preventable by targeting these factors. Abbreviations: CI = confidence interval; CKD = chronic kidney disease; COPD = chronic obstructive pulmonary disease; COVID-19 = coronavirus disease 2019; CVD = cardiovascular disease; LTC = long-term care; NA = not applicable.
